# Role of diversification rates and evolutionary history as a driver of plant naturalization success

**DOI:** 10.1111/nph.17014

**Published:** 2020-11-20

**Authors:** Bernd Lenzner, Susana Magallón, Wayne Dawson, Holger Kreft, Christian König, Jan Pergl, Petr Pyšek, Patrick Weigelt, Mark van Kleunen, Marten Winter, Stefan Dullinger, Franz Essl

**Affiliations:** ^1^ Department of Botany and Biodiversity Research University of Vienna Rennweg 13 Vienna 1030 Austria; ^2^ Instituto de Biología Universidad Nacional Autónoma de México Circuito Exterior, Ciudad Universitaria, Coyoacán Mexico City 04510 Mexico; ^3^ Department of Biosciences Durham University South Road Durham DH1 3LE UK; ^4^ Biodiversity, Macroecology and Biogeography University of Goettingen Büsgenweg 1 Göttingen 37077 Germany; ^5^ Centre of Biodiversity and Sustainable Land Use (CBL) University of Goettingen Büsgenweg 1 Göttingen 37077 Germany; ^6^ Institute for Biochemistry and Biology University of Potsdam Potsdam Germany; ^7^ Institute of Botany Department of Invasion Ecology Czech Academy of Sciences Průhonice CZ‐252 43 Czech Republic; ^8^ Department of Ecology Faculty of Science Charles University Viničná 7 Prague CZ‐128 44 Czech Republic; ^9^ Centre for Invasion Biology Department of Botany & Zoology Stellenbosch University Matieland 7602 South Africa; ^10^ Ecology University of Konstanz Universitätsstrasse 10 Konstanz 78457 Germany; ^11^ Zhejiang Provincial Key Laboratory of Plant Evolutionary Ecology and Conservation Taizhou University Taizhou 318000 China; ^12^ German Centre for Integrative Biodiversity Research (iDiv) Halle‐Jena‐Leipzig Deutscher Platz 5e Leipzig 04103 Germany

**Keywords:** alien species, evolution, geographic distribution, invasion success, plant naturalization, range size

## Abstract

- Human introductions of species beyond their natural ranges and their subsequent establishment are defining features of global environmental change. However, naturalized plants are not uniformly distributed across phylogenetic lineages, with some families contributing disproportionately more to the global alien species pool than others. Additionally, lineages differ in diversification rates, and high diversification rates have been associated with characteristics that increase species naturalization success. Here, we investigate the role of diversification rates in explaining the naturalization success of angiosperm plant families.
- We use five global data sets that include native and alien plant species distribution, horticultural use of plants, and a time‐calibrated angiosperm phylogeny. Using phylogenetic generalized linear mixed models, we analysed the effect of diversification rate, different geographical range measures, and horticultural use on the naturalization success of plant families.
- We show that a family's naturalization success is positively associated with its evolutionary history, native range size, and economic use. Investigating interactive effects of these predictors shows that native range size and geographic distribution additionally affect naturalization success. High diversification rates and large ranges increase naturalization success, especially of temperate families.
- We suggest this may result from lower ecological specialization in temperate families with large ranges, compared with tropical families with smaller ranges.

## Introduction

The introduction of species outside their natural range via human agency has become one of the defining features of recent global environmental change (Crutzen, 2006; Lewis & Maslin, 2015). Unsurprisingly, alien species are not uniformly distributed across the globe or across phylogenetic lineages (Dawson *et al*., 2017; Pyšek *et al*., 2017). Much research has focused on explaining the geographic patterns of alien species richness and their underlying drivers (Lambdon *et al*., 2008; van Kleunen *et al*., 2015a; Capinha *et al*., 2017; Dawson *et al*., 2017; Dyer *et al*., 2017; Pyšek *et al*., 2017), but phylogenetic patterns in biological invasion have received less attention (but see Cadotte *et al*., 2006; Winter *et al*., 2009; Ricotta *et al*., 2012; Fridley & Sax, 2014).

For vascular plants, some families are known to contribute disproportionately more species to the global alien species pool than others (Daehler, 1998; Pyšek, 1998; Pyšek *et al*., 2017). However, using absolute species numbers does not account for the large variation in species richness among families, and consequently does not show the whole picture regarding the naturalization success of individual families (Daehler, 1998; Pyšek, 1998; Pyšek *et al*., 2017). When the total number of species in a family is taken into account, some widespread and species‐rich families (e.g. Poaceae, Fabaceae, Rosaceae) still emerge as contributing a higher number of alien species to the global naturalized alien plant species pool than expected, whereas others contribute proportionally (e.g. Asteraceae) or significantly less than expected (including similarly large families, such as Orchidaceae or Rubiaceae) (Daehler, 1998; Pyšek *et al*., 2017). Moreover, certain clades add disproportionately to the global pool of naturalized alien species, namely the Commelinidae clade (*sensu* Cantino *et al*., 2007), the Fagales, Rosales, and Fabales within Fabidae, and the Alismatales (Pyšek *et al*., 2017). This suggests that the naturalization success of alien species may be associated with their evolutionary history.

The naturalization success of different plant families may increase via phylogenetically clustered traits (Baker, 1974; Theoharides & Dukes, 2007; van Kleunen *et al*., 2010, 2015b; Drenovsky *et al*., 2012). However, families may also differ in their adaptability to novel environments (e.g. to anthropogenic habitats; see Kueffer & Daehler, 2009; Kalusová *et al*., 2017; Otto, 2018), and hence in their ability to successfully establish outside of their native range. On a macroevolutionary scale, adaptation is mediated via diversification, and resulting new species with potential new, more suitable traits and diversification rates can change within regions and within clades. High diversification rates are connected to bursts in species diversity and are often found in tropical regions (especially the Neotropics; Hughes & Eastwood, 2006) or island systems (Baldwin & Sanderson, 1998; Nürk *et al*., 2019), but several studies also identified high diversification rates outside the tropics, such as in the Mediterranean or the Fynbos of South Africa (Linder, 2008; Valente *et al*., 2010). In the tropics, high diversity and high diversification rates coincide in mountain regions like the Northern Andes and are generally thought to result from high environmental heterogeneity and topographical barriers that limit gene flow, cause high potential for species to coexist, occupy vacant niche space, and to persist during periods with fluctuating climatic conditions (Brown, 2014; Rahbek *et al*., 2019). In temperate regions, high diversity is more often attributed to recent and rapid radiations (Linder, 2008; Arakaki *et al*., 2011; Hughes *et al*., 2015). Adaptation to selection pressures under recent and current environmental conditions can imply that species might be able to react more flexibly to changing environmental conditions (including artificial new habitat types) when introduced outside their native range. Hence, differences in diversification rates across lineages might provide valuable insights into the underlying mechanisms that shape global native and alien plant richness.

At the macroevolutionary scale, angiosperm diversification rates differ between families, and within families over time (Sims & McConway, 2003; Soltis & Soltis, 2004). Higher diversification rates have been associated with high species numbers (Sanderson & Donoghue, 1994, 1996; Magallón & Sanderson, 2001). High extant species richness is found in clades with the highest observed diversification rates, with three clades standing out: Commelinids (Poales and Cyperales); Campanulidae (Apiales and Asterales); and Fabidae (Fabales and Rosales) (Magallón & Sanderson, 2001; Sims & McConway, 2003; Magallón & Castillo, 2009). These clades largely coincide with high naturalization success (i.e. families and higher order clades that contribute more naturalized species than expected by chance) across all angiosperms (Pyšek *et al*., 2017). The fact that similar plant clades show high diversification rates as well as high naturalization success strongly suggests that evolutionary history impacts a species’ potential for successful naturalization. However, this relationship and its contribution to explaining large‐scale patterns of alien plant species distributions has never been rigorously examined across the vascular plant phylogeny. We investigate the following two hypotheses involving plant family diversification rates and their naturalization success.

### H1. High diversification rates in plant families are associated with a high naturalization success of the respective families

Based on the congruency between plant family diversification rates and alien species richness per family (as already outlined), we expect a significant positive relationship between the two variables. We consider two additional predictors, mean species range within a family and economic use, to be able to disentangle the role and relative importance of evolutionary history from these other important drivers of naturalization success. Economic use is one major driver of plant invasions (van Kleunen *et al*., 2020), with estimates of 75–93% of naturalized alien plants being cultivated world‐wide (van Kleunen *et al*., 2018). The more individuals of a species (and the more species of a family) are traded and cultivated, the more far‐reaching the dispersal of propagules and the higher the likelihood that the species becomes established outside its native range. Propagule pressure and colonization pressure have been shown to take on a key role in naturalization success (Simberloff, 2009; Blackburn *et al*., 2020), and we thus wanted to statistically control for its effect when analysing the relationship with the evolutionary history of a family.

### H2. High diversification rates for families and small mean tropical or temperate species ranges are associated with a low naturalization success of the respective family

High diversification rates, especially in tropical regions, are associated with higher degrees of (biotic and climatic) specialization and smaller species range sizes (Brown, 2014). High specialization inhibits naturalization success, as the probability of being introduced to a suitable environment is lower than for species with a wider tolerance for environmental factors (Cadotte *et al*., 2006; Richardson & Pyšek, 2012). Smaller ranges, on the other hand, reduce naturalization success, as species are less likely to be displaced unintentionally into a novel environment.

## Materials and Methods

### Data sets

We used five global data sets: (1) The Plant List (The Plant List, 2013), (2) the Global Naturalized Alien Flora (GloNAF) database (Pyšek *et al*., 2017; van Kleunen *et al*., 2019), (3) the Global Inventory of Floras and Traits (GIFT; Weigelt *et al*., 2020), (4) a time‐calibrated angiosperm phylogeny (Magallón *et al*., 2015), and (5) a combined data set of the horticultural use of vascular plants (as used by van Kleunen *et al*., 2018).

The number of extant species in angiosperm families was derived from The Plant List, which is currently the most widely used compilation of taxonomic information for angiosperms. We only used accepted species, excluding those with unassessed or uncertain taxonomic classification. This resulted in a data set of 324 810 species (including infraspecific taxa) belonging to 405 families. We derived the number of naturalized species (i.e. species that form permanent populations outside their native range; Blackburn *et al*., 2011) per family from the GloNAF database v.1.1, which includes 13 138 naturalized species world‐wide belonging to 292 families (van Kleunen *et al*., 2015a). To avoid analytical bias from small families, we excluded those with fewer than 50 species. Global databases, like GloNAF and GIFT, are based on data from a wide range of sources, and, clearly, the availability of plant distribution data differs between different regions of the globe (Meyer *et al*., 2016; Dawson *et al*., 2017). Sampling and data mobilization are often directed more strongly towards specific geographic regions (e.g. North America or Europe) and towards attractive taxonomic groups or clades (Pyšek *et al*., 2008; Meyer *et al*., 2016; Troudet *et al*., 2017). Though this may introduce recording biases in the global data sets we have used, GIFT and GloNAF are the most comprehensive databases on world‐wide native and alien plant species distributions, and we are convinced that potential biases will be modest. Families considered accepted by The Plant List but excluded in this study are listed in Supporting Information Table S1.

The GIFT database (v.1.0) covers 321 563 species from 473 plant families and provides information on their native distribution across 2893 geographic regions. From this data set, we used information for 212 354 angiosperm plant species to calculate mean native species range size per family (hereafter, mean family range). For each individual species, the nonoverlapping region polygons in GIFT containing the species were extracted. We assumed a continuous distribution of the species in all polygons where its presence is recorded in GIFT and the cumulative area (in square kilometres) of all occupied region polygons was used as the species range size. Subsequently, we calculated the mean over all species range sizes within one family (following the aforementioned protocol).

This way of estimating species range sizes, and subsequently the mean family species range, has its limitations of course. First, we likely overestimate the size of the true species range by using the size of the region polygons from GIFT as an estimate of the species range size in that region. This is especially relevant for species with very small native ranges (e.g. endemics), where the range is smaller than the actual polygon included in GIFT. This might lead to a shift in the species distribution of some families towards slightly larger species ranges, subsequently resulting in larger mean species family ranges than be observed in nature. Second, GIFT (as with all global databases) includes data gaps, and multiple regions have different levels of data quality. To assess how spatial completeness of species range information might affect our mean species range estimate, we compared the species range size distribution for each family for three different subsets. The first subset includes all species for the respective family in GIFT, the second includes only those species in GIFT with global distribution information, and the third includes those species in GIFT for which only part of the global distribution range is covered (the number of species for each subset is given in Table S2). For most families the mean species range size for all species (as used in this study) does not strongly diverge from the mean species range size that only includes the species with global coverage (see Fig. S1).

We additionally distinguished species ranges within major global climatic zones to analyse the effect of the tropical vs nontropical distribution of plant families on their naturalization success. We therefore calculated the tropical and nontropical area based on GIFT regions according to the ecoregion classification described by Dinerstein *et al*. (2017), which is an update on the classification published by Olson *et al*. (2001) and used in Antonelli *et al*. (2015). As in Antonelli *et al*. (2015), we merged the following ecoregions to one tropical region: ‘Tropical and Subtropical Moist Broadleaf Forests’, ‘Tropical and Subtropical Dry Broadleaf Forests’, ‘Tropical and Subtropical Coniferous Forests’, and ‘Tropical and Subtropical Grasslands, Savannas, and Shrublands’ (hereafter referred to as ‘tropical’). The remaining ecoregions form the nontropical region (hereafter referred to as ‘temperate’; see Fig. S2 for the delineations). We also calculated the mean species range size for each family in the tropical and temperate part of its native distribution, in the same way as described earlier (hereafter called mean tropical family range and mean temperate family range).

Net diversification rates per family were calculated with the method‐of‐moments estimator of Magallón and Sanderson (2001) – which requires as input data the stem or crown age of a clade (i.e. angiosperm families in this study) – and its extant species richness. Family extant species richness was obtained from The Plant List (http://www.theplantlist.org/). Ages of families were obtained from Magallón *et al*. (2015). The original tree was dated using 792 extant species, representing nearly 90% of angiosperm families and including 136 carefully justified fossil‐calibrated nodes. The possibility to estimate the crown age of a family depends on including representatives from the two branches that diverge from the family’s deepest node leading to extant species (i.e. the crown node). If a clade is represented by only one species, only its stem age (i.e. the time it diverged from its extant sister group) can be estimated. If a clade is represented by two or more species then, in addition to its stem age, its crown age can be estimated, but only if the species sampled derive from the two branches that diverge from the crown node. If the species sampled derive from only one of these branches, their divergence time will be younger than the crown age of the clade. In the study of Magallón *et al*. (2015), many families were represented only by a single species, and for those that were represented by two or more species, it is not guaranteed that the species sampled span the crown node of the family; see Magallón *et al*. (2015) for a more detailed explanation. Thus, the estimates in Magallón *et al*. (2015) provide reliable estimates of angiosperm family stem ages, but not necessarily for crown ages. For this reason, we only considered 315 families from those provided in Magallón *et al*. (2015).

Diversification rate estimates were thus obtained with the method‐of‐moments estimator for stem clades (Magallón & Sanderson, 2001, Eqn 6). The method‐of‐moments estimator can calculate the net rate of diversification *r* = *λ* − *μ* (*λ*, speciation; *μ*, extinction), under a given rate of relative extinction (*ε* = *μ*/*λ*). The relative extinction of a clade is typically unknown, but estimates of the rate of diversification can be obtained for extreme but realistic magnitudes of relative extinction (e.g. *ε* = 0.0 and *ε* = 0.9). The real net diversification rate is expected to fall within those two extreme estimates. Empirical evaluations have shown that the magnitude of net diversification for these extreme values of relative extinction are close (Magallón & Sanderson, 2001). In this study, we found that diversification rate estimates for *ε* = 0.0 did not differ markedly from diversification estimates for *ε* = 0.9, and so only the diversification estimates assuming a high relative extinction rate are reported here.

The horticulture data set was compiled based on three individual data sources: Dave's Garden Plant Files (http://davesgarden.com/guides/pf/, accessed 23 March 2016); the Plant Information Online database (https://plantinfo.umn.edu/, accessed 22 November 2017); and the Plant Search database of Botanic Gardens Conservation International (http://www.bgci.org/plant_search.php, accessed 25 May 2016). After taxonomic standardization using The Plant List (The Plant List, 2013), the data set includes 171 864 species that are cultivated in common or botanical gardens. For a more detailed description of the data set, see van Kleunen *et al*. (2018). Based on this data set, we calculated the proportion of horticulturally used species per family by dividing the number of species per family in the horticulture data set by the total number of species in the family based on The Plant List (hereafter referred to as ‘horticultural use’).

We used naturalization success per plant family as the response variable in our data set. This measure was derived by calculating the proportion of naturalized species per family based on GloNAF to the overall number of species per family based on The Plant List. The proportion was then multiplied by the number of regions where each species of the family is naturalized in to characterize invasion success by both the number of species that have successfully naturalized in at least one region and the total size of the area colonized by these species. The final data set for analyses was created by merging all four individual data sets (number of extant species per family, number of naturalized species per family, mean diversification rate per family, proportion of species per family used horticulturally) and including only those families that were covered by all data sets, resulting in 168 families for analysis (see Table S3).

### Statistical analysis

We ran phylogenetic generalized linear mixed models (PGLMMs) to analyse the effect of diversification rate, different geographical range measures (i.e. mean family range, mean tropical family range, and mean temperate family range), and horticultural use on the naturalization success of plant families. We ran three different models with different predictor sets: model 1 included diversification rate, mean family range, and horticultural use; model 2 included diversification rate, mean tropical family range, mean temperate family range, horticultural use, and interaction terms between diversification rate and each range predictor; and model 3 included diversification rate, mean tropical family range, mean temperate family range, horticultural use, and an interaction term between diversification rate and horticultural use. All predictors were transformed (log or square root) if appropriate to improve symmetry across predictor variables and to stabilize variances. Subsequently, predictor variables were standardized to mean = 0 and SD = 1. Collinearity between predictors played a minor role, as the strongest Pearson correlation was −0.42 (see Table S4 for all correlations).

PGLMMs were run using the MCMCglmm() function from the mcmcglmm package (Hadfield, 2010). For each model, we defined weak informative inverse Wishart priors with the elements *V* = 1 and nu = 0.002 following the suggestions in the mcmcglmm package (Hadfield, 2019). Models followed a Gaussian error distribution and were run for 520 000 iterations with a burn‐in of 20 000 iterations and a thinning interval of 100. Each model was run three times, and model fit was tested visually via chain convergence plots and through Gelman–Rubin diagnostics (Gelman & Rubin, 1992). Chain convergence is achieved when there is no visible pattern within the chain convergence plots (i.e. they resemble white noise; Figs S3–S5) and if the multivariate potential scale reduction factor of the Gelman–Rubin diagnostics is below 1.1 (Table S5). To account for phylogenetic relatedness, we included the inverse of the variance–covariance matrix based on the phylogenetic tree by (Magallón *et al*., 2015) as a random effect structure. For model 1, we performed a stepwise model selection procedure for all model predictor combinations, but always including diversification rate. Each model was run separately and compared based on the deviance information criterion (DIC). Models with lower DIC are preferred over models with higher DIC (Table S6).

To assess PGLMM fit and for model result validation purposes, we additionally ran generalized linear mixed models (GLMMs). The fixed‐effect structure was preserved for the different models. Instead of using the phylogenetic tree as a random effect, a higher taxonomic level (i.e. plant orders) was included. All analyses were performed using the statistical software R v.3.4.3. (R Core Team, 2017). GLMMs were run using the lmer() function in the lme4 package (Bates *et al*., 2015) using a Gaussian error distribution. Model fit was assessed visually using diagnostic plots. Model results are comparable to the ones derived by the PGLMM analysis and, therefore, are only reported in the Supporting Information (Table S7).

## Results

**Fig. 1:**
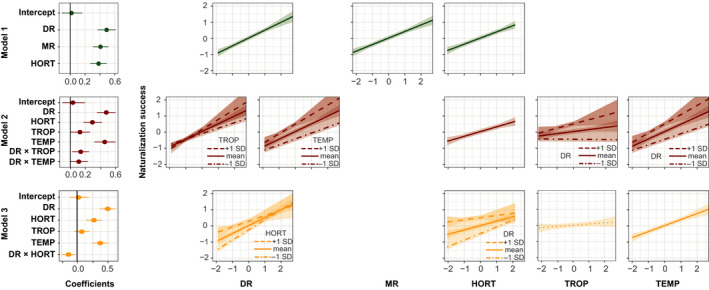
Results of the phylogenetic generalized linear mixed models analysis. Each row provides the coefficient estimates (first column) and marginal effect plots (subsequent columns) for each model. DR, diversification rate; MR, mean family range; HORT, horticultural use; TROP, mean tropical family range; TEMP, mean temperate family range; DR × TROP, interaction term between diversification rate and mean tropical family range; DR × TEMP, interaction term between diversification rate and mean temperate family range; DR × HORT, interaction term between diversification rate and horticultural use. Coefficients are shown with their 95% credibility intervals. In the marginal effects plots, solid lines indicate significant relationships and broken lines nonsignificant relationships. Shaded areas show the 95% credibility intervals.

Naturalization success of plant families was positively related to diversification rate across all models (Fig. 1; Table 1). Diversification rate, mean family range, and horticultural use were positively related to naturalization success (model 1). Diversification rate had the largest effect size (posterior mean = 0.50, *P* < 0.001), followed by mean family range (posterior mean = 0.40, *P* < 0.001), and horticultural use (posterior mean = 0.38, *P* < 0.001).

**Table 1:** Results of the three phylogenetic generalized linear mixed models.

Predictor	Model 1							Model 2							Model 3						
	Posterior mean	95% CI				Eff.samp	*P*	Posterior mean	95% CI				Eff.samp	*P*	Posterior mean	95% CI				Eff.samp	*P*
		Lower		Upper					Lower		Upper					Lower		Upper			
Intercept	0.02	−0.12		0.16		4706	0.764	0.04	−0.11		0.19		4326	0.6148	0.026	−0.13		0.17		4643	0.735
Diversification rate	**0.50**	**0.38**		**0.62**		**1794**	**< 0.0002**	**0.50**	**0.36**		**0.62**		**1711**	**< 0.0002**	**0.50**	**0.39**		**0.63**		**2497**	**< 0.0002**
Mean family range	**0.40**	**0.30**		**0.50**		**4883**	**< 0.0002**														
Horticultural use	**0.38**	**0.28**		**0.50**		**4565**	**< 0.0002**	**0.30**	**0.18**		**0.42**		**5000**	**< 0.0002**	**0.28**	**0.15**		**0.39**		**5000**	**< 0.0002**
Mean tropical family range								**0.13**	**0.00**		**0.25**		**5000**	**0.0388**	0.08	−0.04		0.20		5000	0.218
Mean temperate family range								**0.45**	**0.33**		**0.60**		**5754**	**< 0.0002**	**0.37**	**0.24**		**0.49**		**5235**	**< 0.0002**
DR × TROP								**0.14**	**0.02**		**0.25**		**5000**	**0.0216**							
DR × TEMP								0.11	0.00		0.23		5000	0.0588							
DR × Horticulture															−**0.14**	−**0.25**		−**0.03**		**5000**	**0.010**

CI, credibility interval; Eff.samp, effective number of samples; DR × TROP, interaction term between diversification rate and mean tropical family range; DR × TEMP, interaction term between diversification rate and mean temperate family range; DR × HORT, interaction term between diversification rate and horticultural use.

We ran each model for 520 000 iterations with a burn‐in of 20 000 iterations and a thinning interval of 100. Estimates highlighted in bold indicate significant terms.

Introducing an interaction term between diversification rate and the mean tropical and temperate family range mirrored the results from model 1 in terms of the positive relationships between naturalization success and diversification rate (posterior mean = 0.49, *P* < 0.001) and horticultural use (posterior mean = 0.30, *P* < 0.001). Naturalization success showed a significantly positive increase with mean temperate family range (posterior mean = 0.45, *P* < 0.001) and a weak but significant increase with increasing mean tropical family range (posterior mean = 0.13, *P* = 0.039). Interaction terms between diversification rate and mean tropical range showed a weak significant positive trend (posterior mean = 0.14, *P* = 0.022), indicating that the effect of diversification rate on naturalization success is strongest for families with large mean tropical range size. There was no interaction between diversification rate and mean temperate family range (posterior mean = 0.11, *P* = 0.059).

Finally, in model 3, we analysed the interaction effect between diversification rate and horticultural use. Single predictor trends remained significantly positive for diversification rate (posterior mean = 0.50, *P* < 0.001), mean temperate family range (posterior mean = 0.37, *P* < 0.001), and horticultural use (posterior mean = 0.28, *P* < 0.001). The significant negative interaction term (posterior mean = −0.14, *P* = 0.010) showed that naturalization success increases more strongly with increasing diversification rates for plant families that are less intensively used in horticulture. Model estimates for all models are shown in Table 1.

## Discussion

In this study, we illustrate the relevance of evolutionary history in explaining the naturalization success for 168 plant families world‐wide. Here, we systematically assess the role of evolutionary history and demonstrate that diversification rates can significantly contribute to the explanation of macroecological patterns of plant naturalization by using plant families, confirming hypothesis H1. This extends previous studies that indicated naturalization success across plant families might be phylogenetically clustered within specific clades (Pyšek, 1998; Pyšek *et al*., 2017). Besides these novel findings, our analyses confirm previous studies suggesting that large native species range sizes increase naturalization success (Cadotte *et al*., 2006; Richardson & Pyšek, 2012) and that high propagule pressure (measured as horticultural use) is a main driver of plant family naturalizations (Dehnen‐Schmutz *et al*., 2007; Simberloff, 2009; van Kleunen *et al*., 2018).

As for most macroecological and macroevolutionary predictors, diversification rate is a proxy for underlying processes that operate at different taxonomic and spatial scales. Thus, diversification rate, as used in this study, is related to different mechanisms relevant for alien plant species naturalizations. For instance, high diversification rates in angiosperm lineages are related to the evolution of specific functional traits or trait states (Vamosi *et al*., 2018; Hernández‐Hernández & Wiens, 2020). Additionally, the evolution of lineage‐specific traits throughout evolutionary history (i.e. synapomorphies) may coincide with an increase in diversification rates across certain families (Vamosi *et al*., 2018). Several such traits are also associated with higher invasion success in alien plants, including fruit size and dispersal mode (i.e. fleshy fruits; Pyšek & Richardson, 2007; Onstein *et al*., 2017), large compound inflorescences (Daehler, 1998; Preston, 2010; Castro *et al*., 2016), and phenotypic plasticity (Ghalambor *et al*., 2007; Hulme, 2008; Pyšek *et al*., 2009; Drenovsky *et al*., 2012; Gallagher *et al*., 2015; Huang *et al*., 2015). Other biological properties, like selfing ability (Razanajatovo *et al*., 2016), dispersal ability (Cheptou *et al*., 2008), maximum plant height (Siemann & Rogers, 2001; Blumenthal & Hufbauer, 2007; Pyšek & Richardson, 2007; van Kleunen *et al*., 2010), ploidy level (e.g. polyploidy; Te Beest et al., 2012), or seed longevity (Gioria *et al*., 2012; Pyšek *et al*., 2015), have also been identified as traits determining invasion success. Moreover, the evolution of attributes that result in greater adaptation to new environmental conditions was associated with high diversification rates in several of the 10 families with highest naturalization success (Table S3). This includes the repeated evolution of the C_4_ photosynthesis pathway in Poaceae in response to palaeoclimatic changes (Osborne, 2008; Spriggs *et al*., 2014) or the evolution of nodulation in Fabaceae as adaptation to semiarid areas and in response to high atmospheric CO_2_ concentrations (Sprent & James, 2007). The evolution of traits related to naturalization success under current climates and the short‐term adaptation to novel environmental conditions may thus also promote biological invasions and shape patterns of evolutionary history in plant family naturalization success.

The positive interaction between diversification rates and mean tropical family range supports our hypothesis H2. Naturalization success is higher for families with high diversification rates and large mean species range in the tropics, compared with those with high diversification rates and small mean species ranges in the tropics (model 2; Fig. 1; Table 1). Hence, high diversification rates may be associated with biological characteristics that result in lower naturalization success. Especially in the tropics, high diversification rates tend to result in a higher degree of specialization through adaptive radiation (Klopfer & MacArthur, 1961; Brown, 2014) or the evolution of strong mutualistic interactions (e.g. pollination syndromes, plant–plant or plant–fungi interactions). These evolutionary adaptations generally coincide with restriction to narrow environmental niches and limit range expansion (Goodwin *et al*., 1999; Pyšek *et al*., 2009). Should a species, nevertheless, be transported to a region outside of its native range, this high degree of specialization likely reduces the probability of establishment; for example, due to the absence of obligate mutualists (e.g. mycorrhizal fungi or specialist pollinators; Mitchell *et al*., 2006; but see Richardson *et al*., 2000) or the ability to cope with specific abiotic habitat characteristics. Finally, tropical and subtropical species generally have narrower climatic niches (Rapoport, 1982; Brown *et al*., 1996, 2014) that further constrain the area they can colonize. As an example, climatic filters, like frost events, limit the establishment of species from tropical families; and vice versa, species from temperate families might tolerate tropical conditions or find suitable habitats in tropical mountain regions that are climatically more similar to their native ranges. In some cases, especially for tropical montane species, these constraints might be less severe. In fact, climatic niche conservatism in the tropics might be asymmetrical (Smith *et al*., 2012), with montane species being more flexible and adaptable given their more recent evolution during the Pleistocene (Donoghue, 2008; Smith *et al*., 2012; Kerkhoff *et al*., 2014). Consequently, naturalization potential based on climatic adaptability among these families might be higher than in lowland tropical families with tighter climatic niches. At the same time, tropical mountain species usually have smaller ranges than temperate ones, which reduces the likelihood of species being picked up and transported outside their native range.

Here, we provide a coarse regional subdivision into tropical and subtropical ecoregions to calculate mean tropical and temperate family range sizes, which does not account for intra‐ecoregion variation and the respective complexity in evolutionary dynamics at a finer scale. Microclimates, as well as local differences in other environmental conditions (e.g. water and nutrient availability), are known to shape evolutionary processes through local adaptation and specialization (e.g. in tropical mountains or in arid systems and at higher latitudes; Rahbek *et al*., 2019; Ramírez‐Barahona *et al*., 2020). Unfortunately, calculating range sizes for smaller entities from regional checklists is impossible, or would require strong assumptions and hence provide unreliable results. Future studies should concentrate on different biomes (e.g. the Mediterranean) and regions (e.g. tropical mountain ranges) with high‐resolution species occurrence data to investigate the importance of small‐scale evolutionary dynamics on the naturalization success of angiosperm plants.

Besides evolutionary and ecological reasons, socioeconomic factors and human history strongly influence the geographic distribution of alien species world‐wide. Horticulture is a major driver of transporting plant species across the world (Bradley *et al*., 2012; Mayer *et al*., 2017; van Kleunen *et al*., 2018, 2020). Horticultural use significantly increases naturalization success across all models, although it never emerges as the strongest predictor, and the negative interaction between diversification rate and horticultural use (model 3; Fig. 1; Table 1) indicates that with increased horticultural use, which can be understood as a proxy for introduction effort and propagule and colonization pressure, the evolutionary history of a plant family becomes less important.

Finally, our models only show a weak significant (model 2; Fig. 1; Table 1) and a nonsignificant (model 3; Fig. 1; Table 1) positive relationship of mean tropical family range and naturalization success but a quite strong positive relationship for mean temperate family range and naturalization success (models 2 and 3; Fig. 1; Table 1). This stronger relationship for families with large mean temperate ranges supports previous findings of higher alien plant species richness in temperate regions than in tropical ones (van Kleunen *et al*., 2015a; Turbelin *et al*., 2017). This finding might be explained by historical geopolitical power structures (i.e. European colonization and colonial empires; di Castri, 1989), associated trade networks (Chapman *et al*., 2017), the distribution of botanical gardens (Hulme 2011), and differences in historic and current socio‐economic development (Turbelin *et al*., 2017) – all factors that influence the likelihood that species are picked up, transported, and introduced to new regions where they may successfully naturalize. The underrepresentation of mainly tropical and subtropical regions of the Southern Hemisphere from the global economic trade network might have strongly discriminated against tropical and subtropical families in the global alien plant species pool (van Kleunen *et al*., 2015a). Here, we find, on average, more alien species in temperate families than in tropical ones (Table S3). Among the most successful ones, many contain therophytes (*sensu* Raunkiær, 1934), or annual species that are well adapted to ruderal conditions (e.g. Amaranthaceae, Convolvulaceae, Cyperaceae, Papaveraceae, Polygonaceae); this life history strategy has been shown to promote the naturalization of alien plants (Guo *et al*., 2018). Another example are the members of the Fabaceae family, which vary widely in growth form (from small ephemeral species to large forest trees), pollination, or dispersal mechanism, but share the ability to fix atmospheric nitrogen, which makes them especially successful in coping with novel environments in their introduced range (Heywood, 1989; Daehler, 1998). Finally, some of the most successful naturalized plant families are associated with high economic importance for either agricultural or horticultural use (e.g. Amaranthaceae, Fabaceae, Rosaceae; Daehler, 1998; van Kleunen *et al*., 2018). Among the least successful families in our study are exclusively or mostly tropically distributed families, most of which consist of woody species (e.g. Vochysiaceae, Stemonuraceae, Siparunaceae, Sabiaceae, or Picrodendraceae). Species of other families occur in mainly small, sometimes disjunct ranges (e.g. Bruniaceae, Restionaceae); and among all unsuccessful families, generally few species are used horticulturally (e.g. *Norantea guianensis* in Marcgraviaceae or *Calopsis paniculata* in Restionaceae).

Our study contributes to the identification of indicators explaining large‐scale macroevolutionary patterns, with a special focus on the relationship between family naturalization success and family diversification rates. Here, we quantify diversifications rates using the method‐of‐moments estimator (Magallón & Sanderson, 2001) that provides mean diversification rates for the families considered. As pointed out earlier, substantial progress has been made towards estimating the change of diversification rates through time (e.g. Stadler, 2011; Rabosky, 2014; Morlon *et al*., 2016). Such data would undoubtedly provide additional interesting information into this relationship. Unfortunately, all of these methods require a species‐level phylogeny, which currently is not available at the taxonomic extent of our study. A species‐level phylogeny including all families in this study would need to have 277 824 species tips. The most comprehensive tree to date based on sequence data includes just 79 881 species (Smith & Brown, 2018).

Estimation of diversification parameters (speciation and extinction, or diversification and relative extinction) is complicated because accurate information of the rate of extinction is unavailable from extant diversity or is available with variable degrees of incompleteness for different clades in the fossil record. Available parametric methods that investigate diversification dynamics – for example, whole‐tree diversification changes through time (Stadler, 2011; Morlon *et al*., 2011) or diversification shifts among phylogenetic branches (e.g. Rabosky 2014, May *et al*., 2016) – are not focused on directly estimating diversification rate parameters (Sanmartín & Meseguer, 2016). In this study, we used a method‐of‐moments estimator (Magallón & Sanderson, 2001) of the per‐family rate of diversification. This estimator is based on a stochastic birth–death model that explicitly accounts for extinction and can provide estimates considering the stem age or crown age of a clade. Nevertheless, it has two important caveats: it requires that the rate of relative extinction is known, and it treats the diversification process as time homogeneous. For each family, we estimated the rate of diversification under two extreme, but realistic magnitudes of relative extinction: *ε* = 0.0, which corresponds to zero extinction, and *ε* = 0.9, in which the rate of extinction is close to, but lower than, the rate of speciation. We somewhat arbitrarily chose this upper bound because, at higher relative extinction rates, in particular, ≥ 1, the probability of survival of a clade to the present becomes very small, and diversification becomes a chaotic process dominated by stochastic extinction and extremely rapid turnover (Magallón & Sanderson, 2001). In congruence with previous results, we found here that per‐family diversification rates estimated under the two bounds were similar, and hence, we used only those estimated assuming *ε* = 0.9.

### Conclusions

Our assessment of the naturalization success of plant families in relation to their evolutionary history reveals that high diversification rates are positively related to naturalization success of families. Further, our results indicate an interaction between the evolutionary history and propagule pressure, where evolutionary history becomes less important with an increase in the economic use of a family. With respect to the geographic distribution, tropical families with small ranges and high diversification rates are less successful in naturalizing than families in tropical regions with larger mean species ranges are. We argue that both natural and socio‐economic processes contribute to the observed relationship between diversification rates, mean species range size, and naturalization success. Though ecological characteristics linked to the evolutionary history of a family will more likely affect the establishment and spread phase of alien species, the geographic distribution of the species in a family, in conjunction with socio‐economic processes, underpins the initial displacement and introduction phase of alien species. Our findings support the notion that certain ecological characteristics constrain invasion success and will continue to do so (e.g. high degrees of specialization). On the contrary, tropical and subtropical families with high diversification rates and low specialization might become more important in the future pool of alien species as a consequence of the restructuring of global trade networks towards emerging economies (which are mainly located in (sub)tropical regions) and the intensification of global trade.

## Author contributions

BL, FE and SD designed the study; BL performed all analysis; SM, HK, PW, CK and MvK provided substantial amounts of data; BL led the writing with substantial input from SM, WD, MW, PP, HK, CK, PW, JP, SD, FE and MvK to revisions. SD and FE contributed equally to this work.

## Supporting information


**Fig. S1** Density plots for the calculated species range sizes per family based on data from the GIFT database.
**Fig. S2** World map including the non‐overlapping regions used to calculate range estimates in the analysis.
**Fig. S3** Diagnostic plots to assess chain convergence for the different PGLMM runs for model 1.
**Fig. S4** Diagnostic plots to assess chain convergence for the different PGLMM runs for model 2.
**Fig. S5** Diagnostic plots to assess chain convergence for the different PGLMM runs for model 3.
**Table S1** Families considered as accepted by The Plant List and that have been excluded from the analysis.
**Table S2** Overview of the number of species records for each family and used within the study to calculate mean species range sizes.
**Table S3** Full dataset used in the analysis including 168 plant families.
**Table S4** Pearson correlation between all predictor variable combinations.
**Table S5** Gelman‐Rubin diagnostic to assess chain convergence for all three PGLMM models.
**Table S6** Model selection results for model 1.
**Table S7** Results of the three generalized linear mixed models.
Please note: Wiley Blackwell are not responsible for the content or functionality of any Supporting Information supplied by the authors. Any queries (other than missing material) should be directed to the *New Phytologist* Central Office.

## Data Availability

Data used to perform the analysis in this manuscript are correctly cited in case the databases are published. All other data or derived variables are provided in the supplementary material.
