# Supplementary material for: Role of diversification rates and evolutionary history as a driver of plant naturalization success
Source: New Phytol. 2020 Nov 20;229(5):2998–3008. doi: 10.1111/nph.17014 (PMC7894487; doi:10.1111/nph.17014)
Supplement: Supplementary file 1 — Fig. S1 Density plots for the calculated species range sizes per family based on data from the GIFT database. Fig. S2 World map including the non‐overlapping regions used to calculate range estimates in the analysis. Fig. S3 Diagnostic plots to assess chain convergence for the different PGLMM runs for model 1. Fig. S4 Diagnostic plots to assess chain convergence for the different PGLMM runs for model 2. Fig. S5 Diagnostic plots to assess chain convergence for the different PGLMM runs for model 3. Table S1 Families considered as accepted by The Plant List and that have been excluded from the analysis. Table S2 Overview of the number of species records for each family and used within the study to calculate mean species range sizes. Table S3 Full dataset used in the analysis including 168 plant families. Table S4 Pearson correlation between all predictor variable combinations. Table S5 Gelman‐Rubin diagnostic to assess chain convergence for all three PGLMM models. Table S6 Model selection results for model 1. Table S7 Results of the three generalized linear mixed models. Please note: Wiley Blackwell are not responsible for the content or functionality of any Supporting Information supplied by the authors. Any queries (other than missing material) should be directed to the New Phytologist Central Office. [file NPH-229-2998-s001.pdf]

New Phytologist Supporting Information

Article title: The role of diversification rates and evolutionary history as a driver of plant naturalization success

Authors: Bernd Lenzner, Susana Magallón, Wayne Dawson, Holger Kreft, Christian König, Jan Pergl, Petr Pyšek, Patrick Weigelt, Mark van Kleunen, Marten Winter, Stefan Dullinger and Franz Essl

Article acceptance date: 12 October 2020

Fig. S1: Density plots for the calculated species range sizes per family based on data from the GIFT database. Each panel provides information for one of the 168 families used in this study. The bold, black density distribution shows the species range distribution for all species included in GIFT. The blue distribution curve provides the species range distribution for only those species where GIFT included information on their global distribution range and the red distribution curve gives the species range distribution for those species where GIFT does not cover the entire species range. Vertical lines show the mean (solid) and median (dashed) of the respective density distributions. For families where less than 5 species have either a global or non-global distribution, only the overall species range size distribution is provided. For more information on how the ranges were calculated refer to the methods section of the manuscript. Information about how many species have a global or non-global coverage in GIFT please refer to table S5.

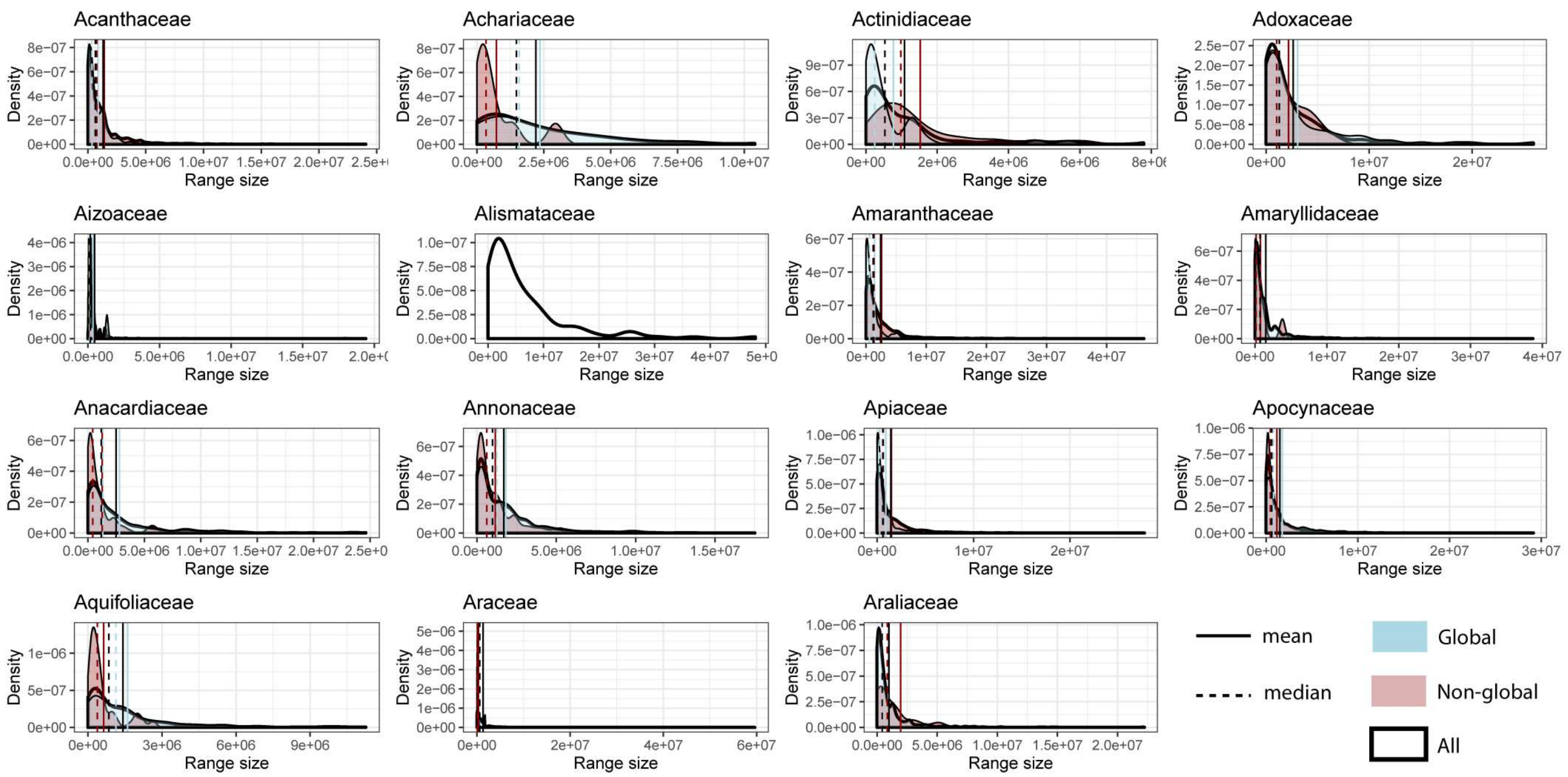

Acanthaceae
Achariaceae
Actinidiaceae
Adoxaceae
Aizoaceae
Alismataceae
Amaranthaceae
Amaryllidaceae
Anacardiaceae
Annonaceae
Apiaceae
Apocynaceae
Aquifoliaceae
Araceae
Araliaceae
Density
Range size
mean
median
Global
Non-global
All

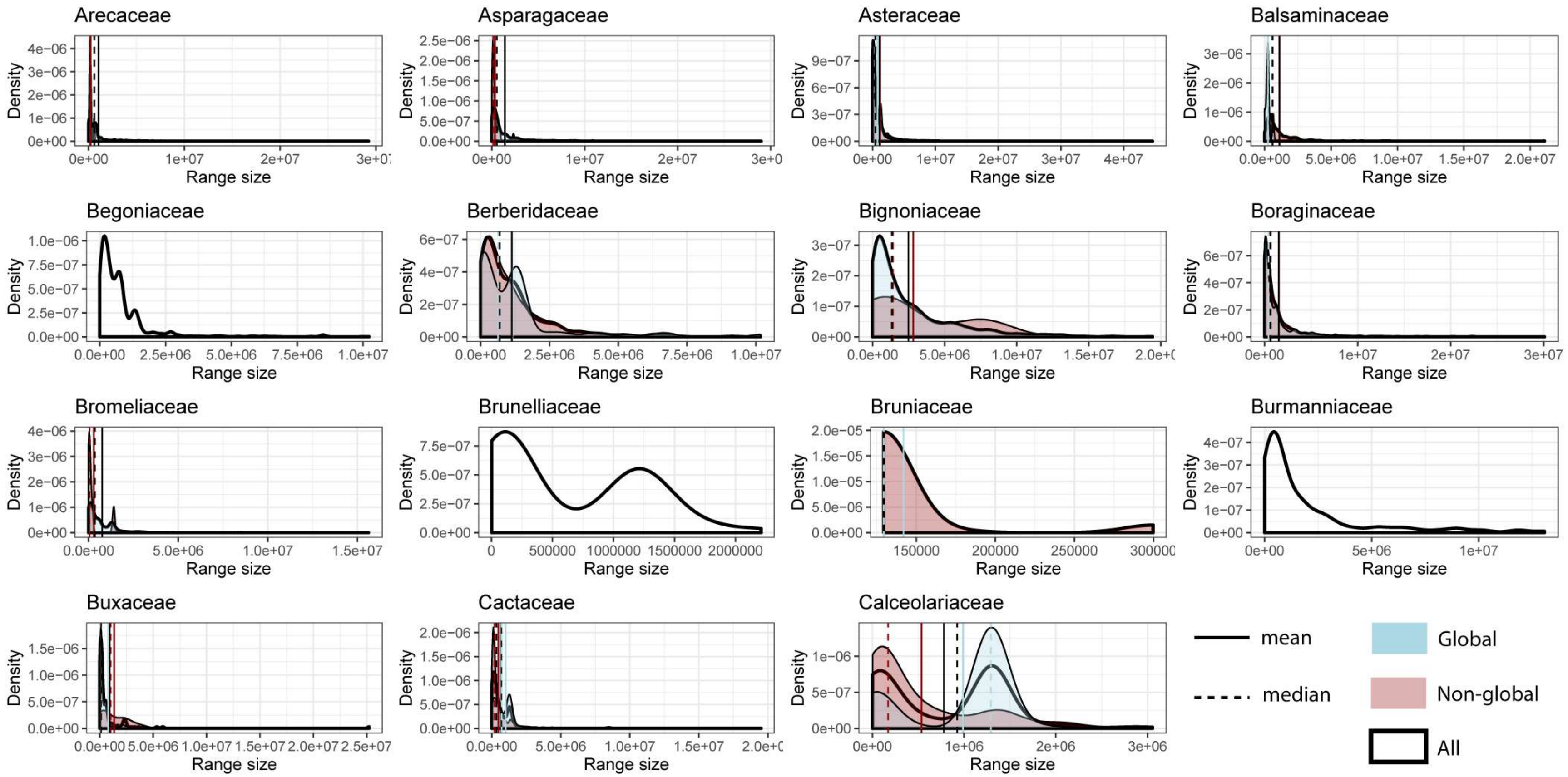

Arecaceae
Asparagaceae
Asteraceae
Balsaminaceae
Begoniaceae
Berberidaceae
Bignoniaceae
Boraginaceae
Bromeliaceae
Brunelliaceae
Bruniaceae
Burmanniaceae
Buxaceae
Cactaceae
Calceolariaceae
Density
Range size
mean
median
Global
Non-global
All

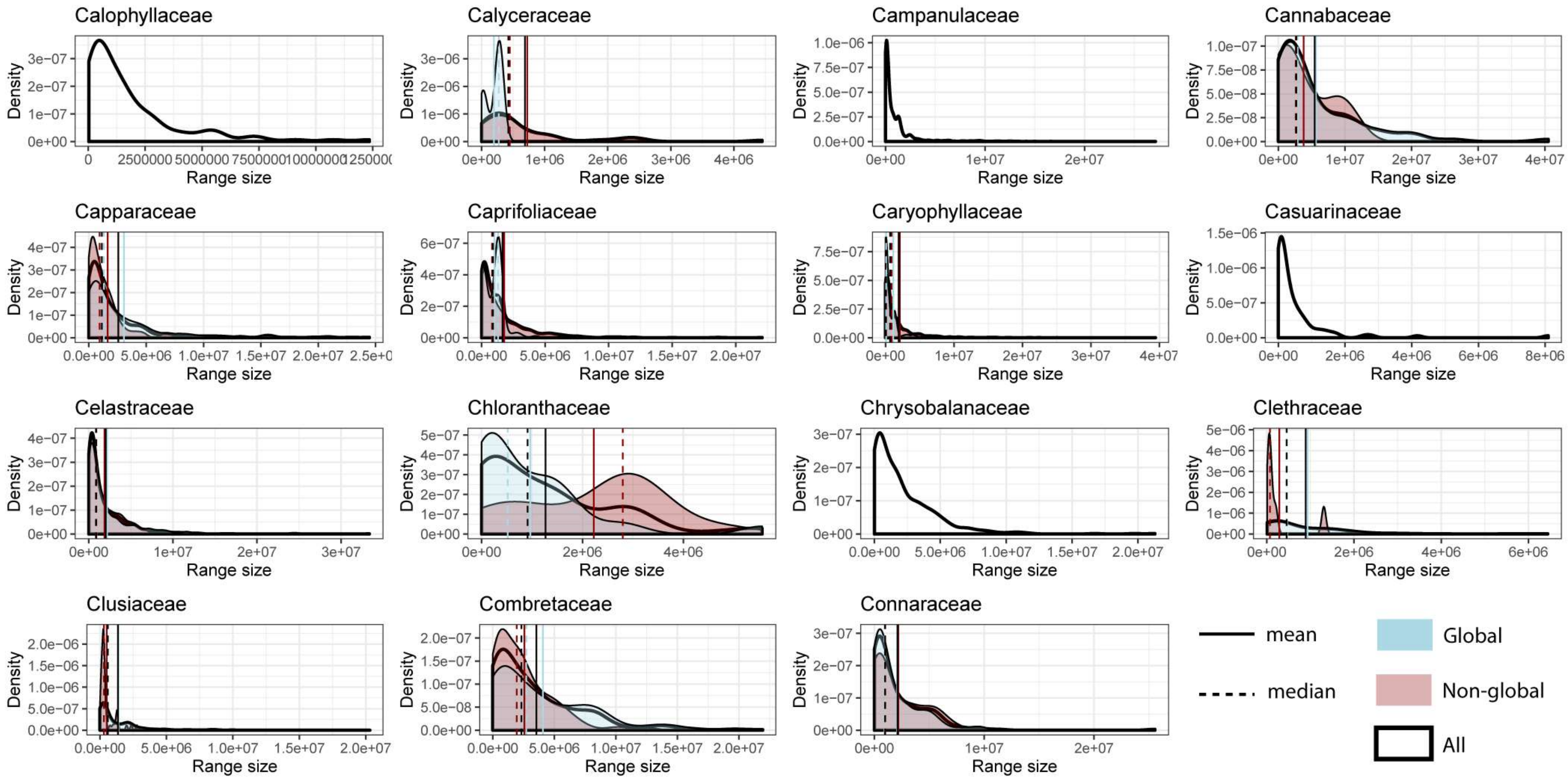
Calophyllaceae
Calyceraceae
Campanulaceae
Cannabaceae
Capparaceae
Caprifoliaceae
Caryophyllaceae
Casuarinaceae
Celastraceae
Chloranthaceae
Chrysobalanaceae
Clethraceae
Clusiaceae
Combretaceae
Connaraceae
Density
Range size
mean
median
Global
Non-global
All

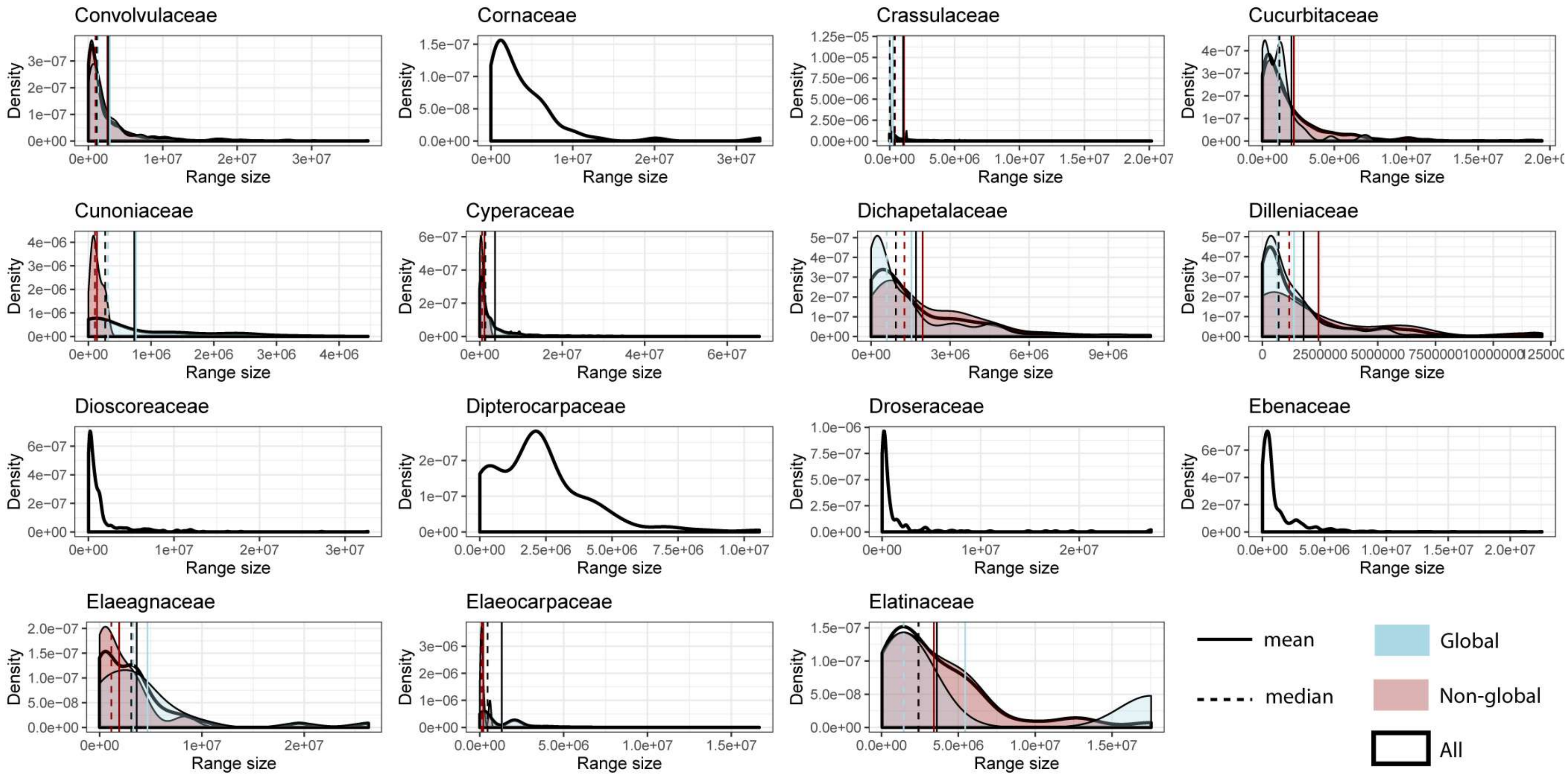
Convolvulaceae
Cornaceae
Crassulaceae
Cucurbitaceae
Cunoniaceae
Cyperaceae
Dichapetalaceae
Dilleniaceae
Dioscoreaceae
Dipterocarpaceae
Droseraceae
Ebenaceae
Elaeagnaceae
Elaeocarpaceae
Elatinaceae
Density
Range size
mean
median
Global
Non-global
All

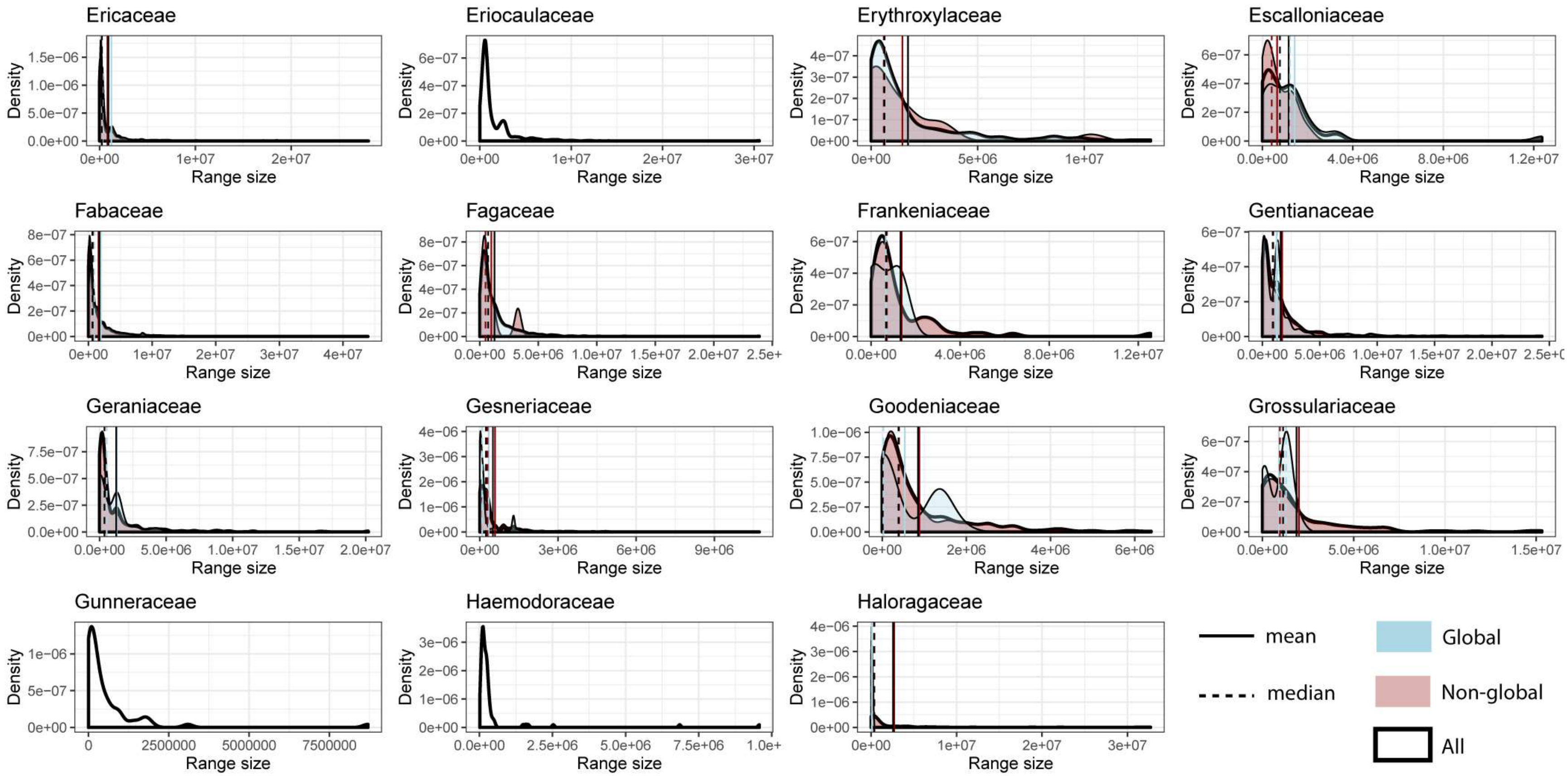

Ericaceae
Eriocaulaceae
Erythroxylaceae
Escalloniaceae
Fabaceae
Fagaceae
Frankeniaceae
Gentianaceae
Geraniaceae
Gesneriaceae
Goodeniaceae
Grossulariaceae
Gunneraceae
Haemodoraceae
Haloragaceae
Density
Range size
mean
median
Global
Non-global
All

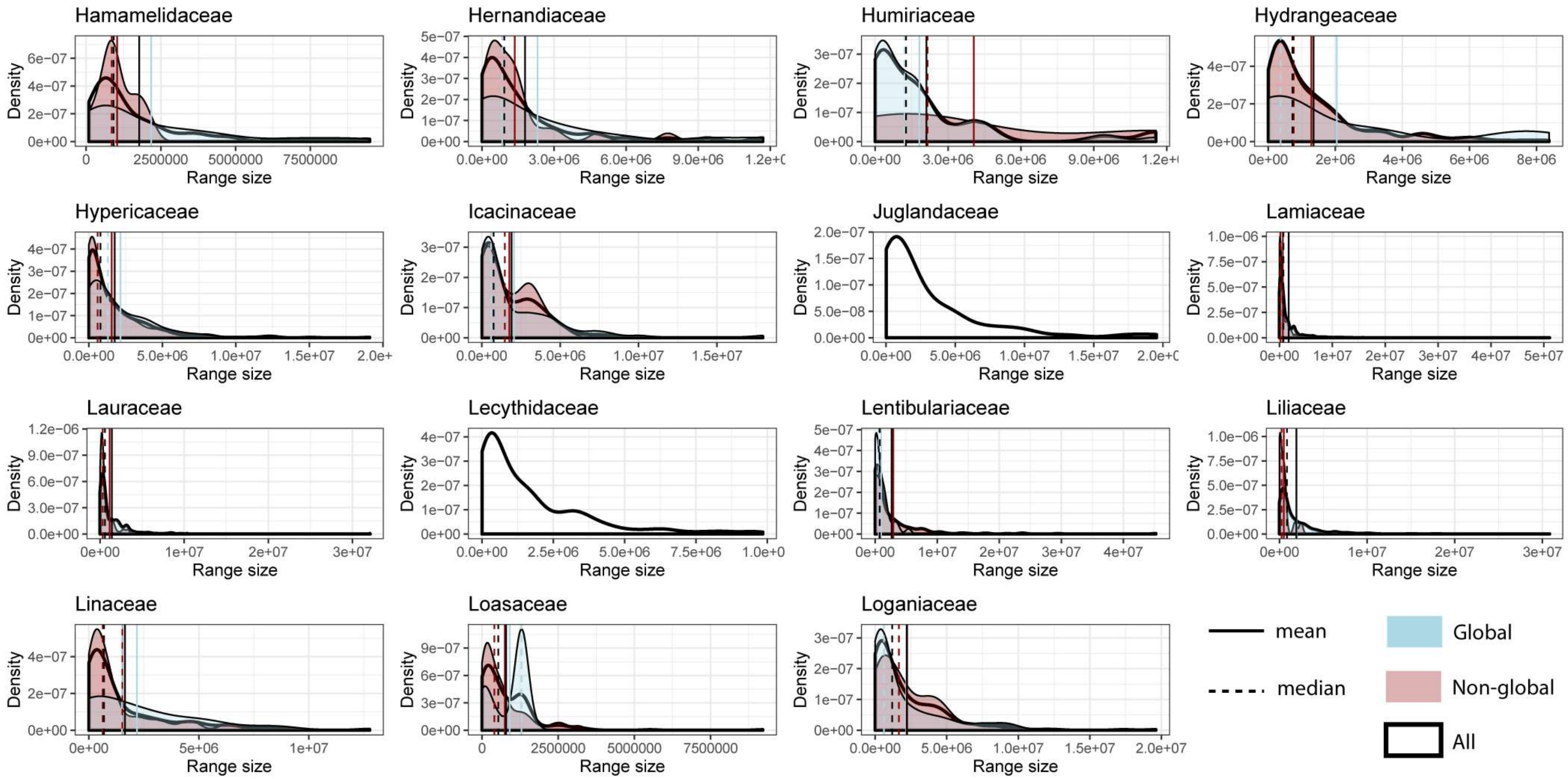
Hamamelidaceae
Hernandiaceae
Humiriaceae
Hydrangeaceae
Hypericaceae
Icacinaceae
Juglandaceae
Lamiaceae
Lauraceae
Lecythidaceae
Lentibulariaceae
Liliaceae
Linaceae
Loasaceae
Loganiaceae
Density
Range size
mean
median
Global
Non-global
All

22

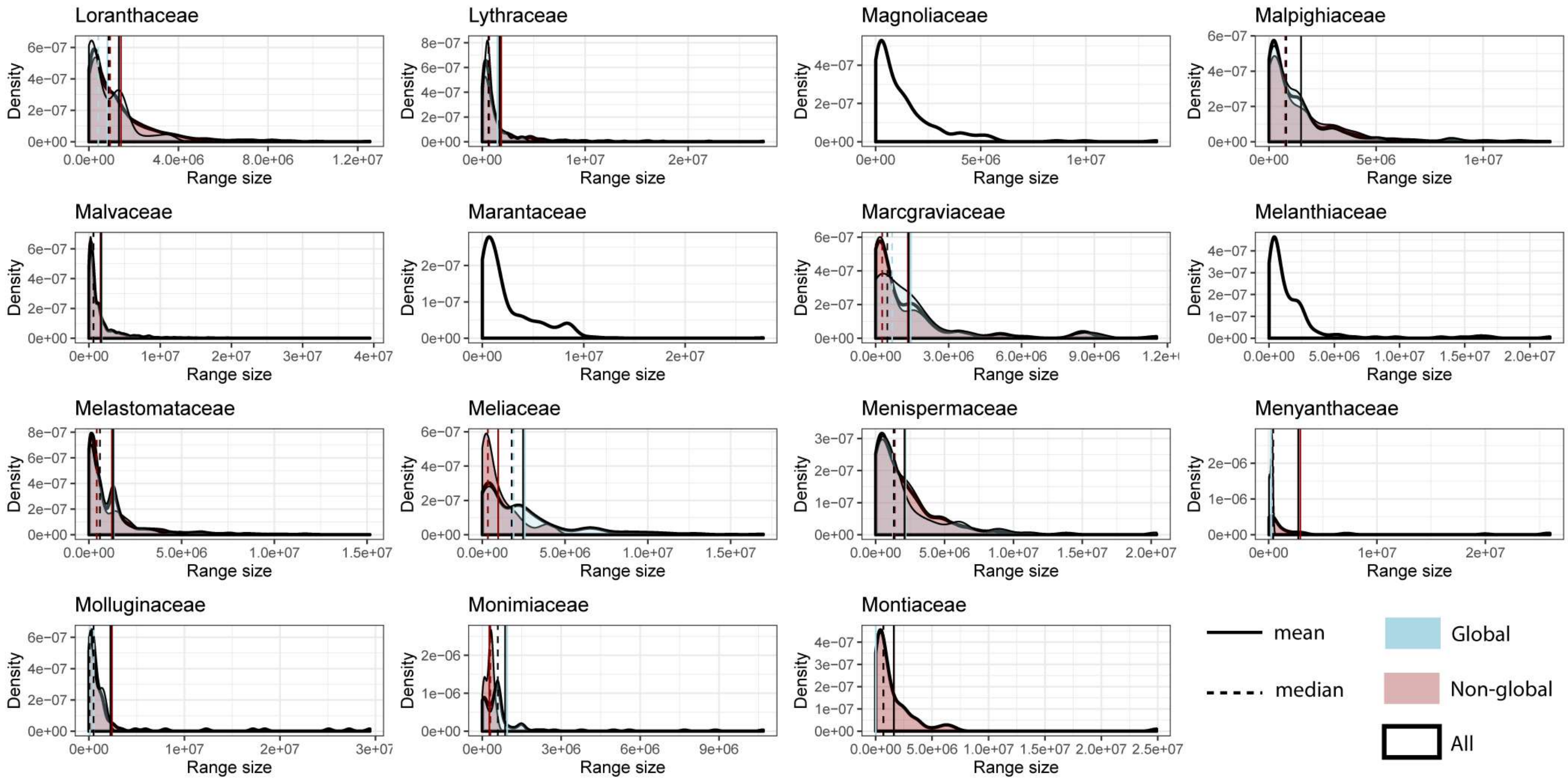
Loranthaceae
Lythraceae
Magnoliaceae
Malpighiaceae
Malvaceae
Marantaceae
Marcgraviaceae
Melanthiaceae
Melastomataceae
Meliaceae
Menispermaceae
Menyanthaceae
Molluginaceae
Monimiaceae
Montiaceae
Density
Range size
mean
median
Global
Non-global
All

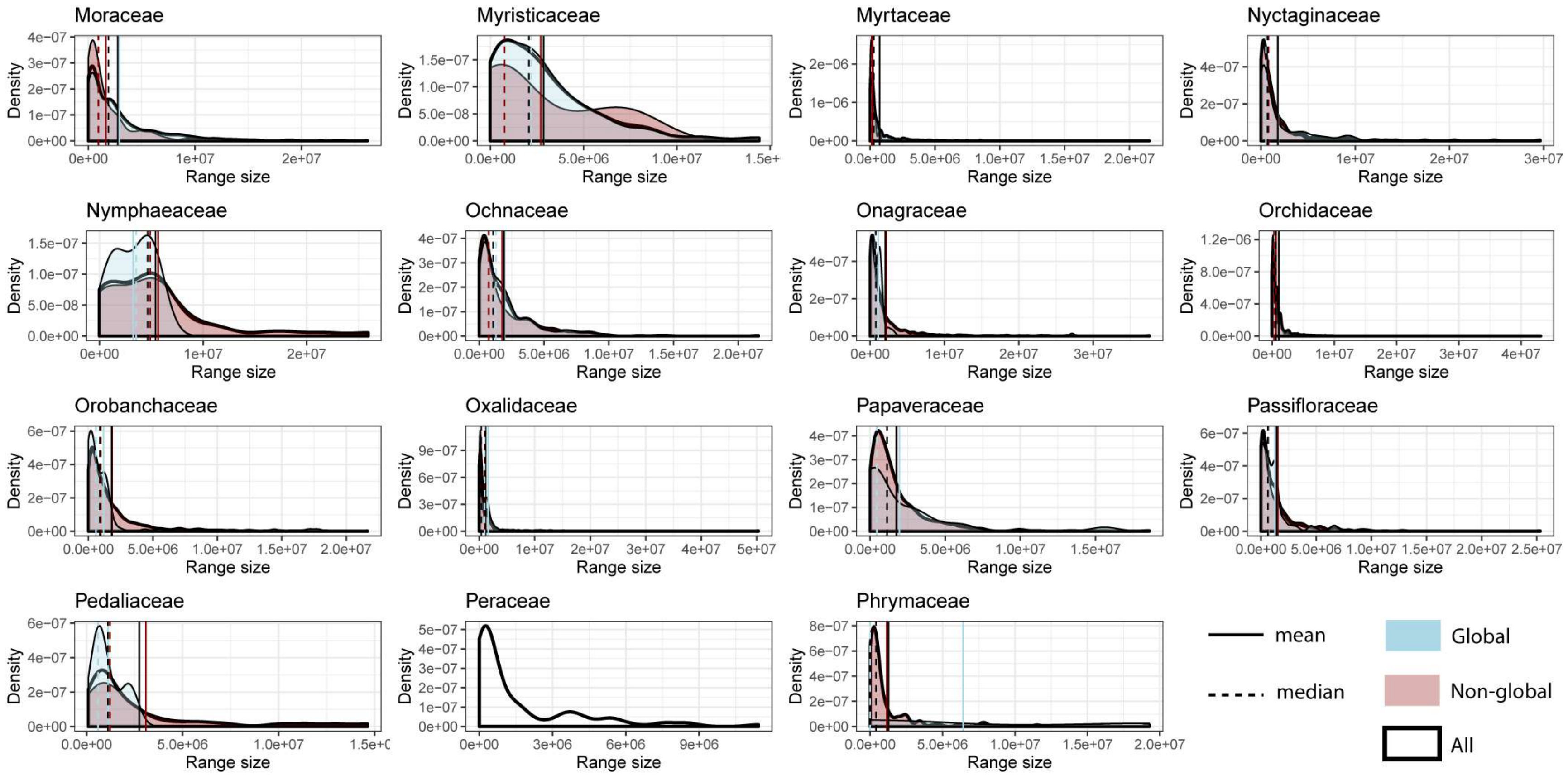
Moraceae
Myristicaceae
Myrtaceae
Nyctaginaceae
Nymphaeaceae
Ochnaceae
Onagraceae
Orchidaceae
Orobanchaceae
Oxalidaceae
Papaveraceae
Passifloraceae
Pedaliaceae
Peraceae
Phrymaceae
Density
Range size
mean
median
Global
Non-global
All

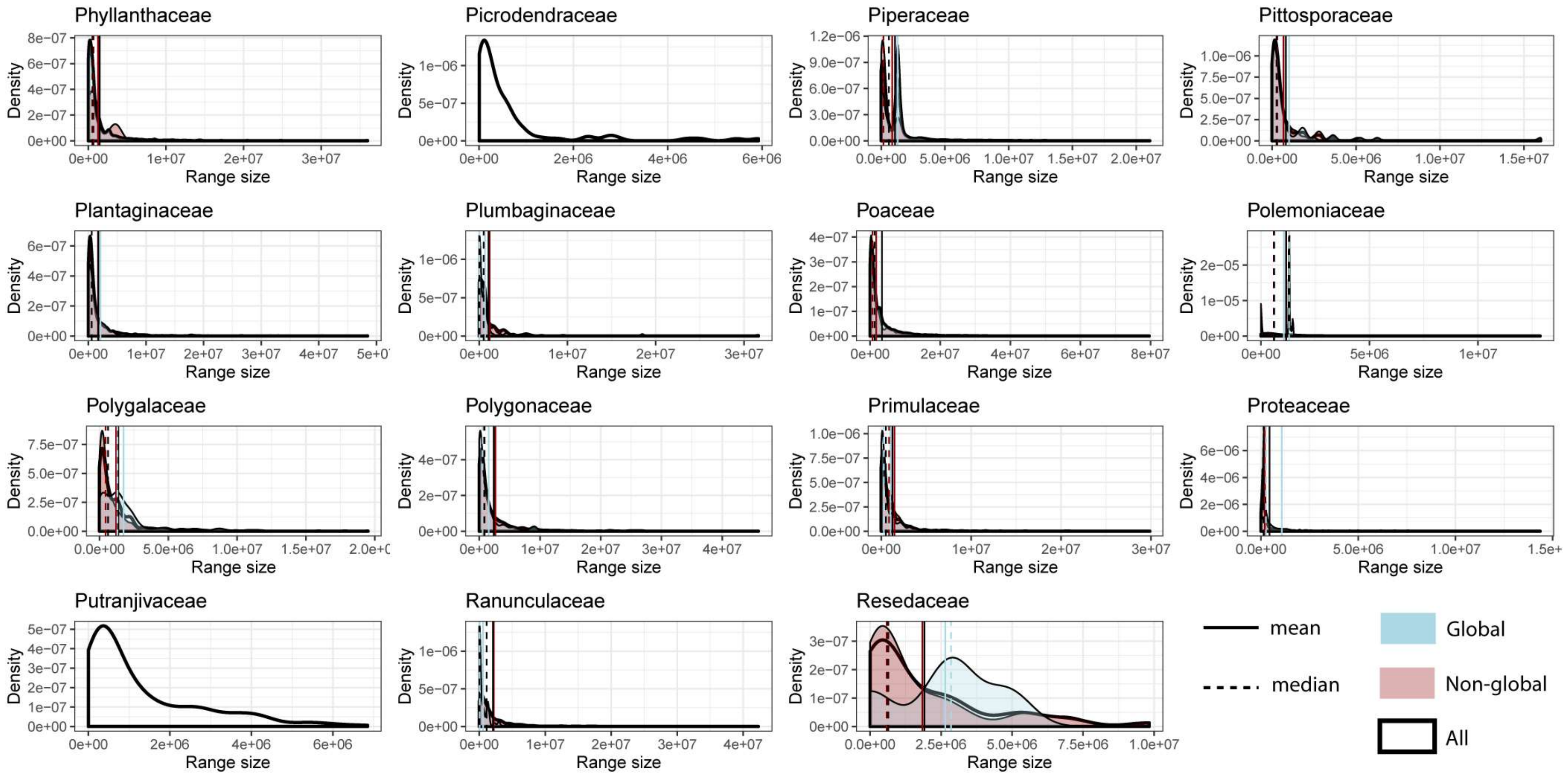
Phyllanthaceae
Picrodendraceae
Piperaceae
Pittosporaceae
Plantaginaceae
Plumbaginaceae
Poaceae
Polemoniaceae
Polygalaceae
Polygonaceae
Primulaceae
Proteaceae
Putranjivaceae
Ranunculaceae
Resedaceae
Density
Range size
mean
median
Global
Non-global
All

25

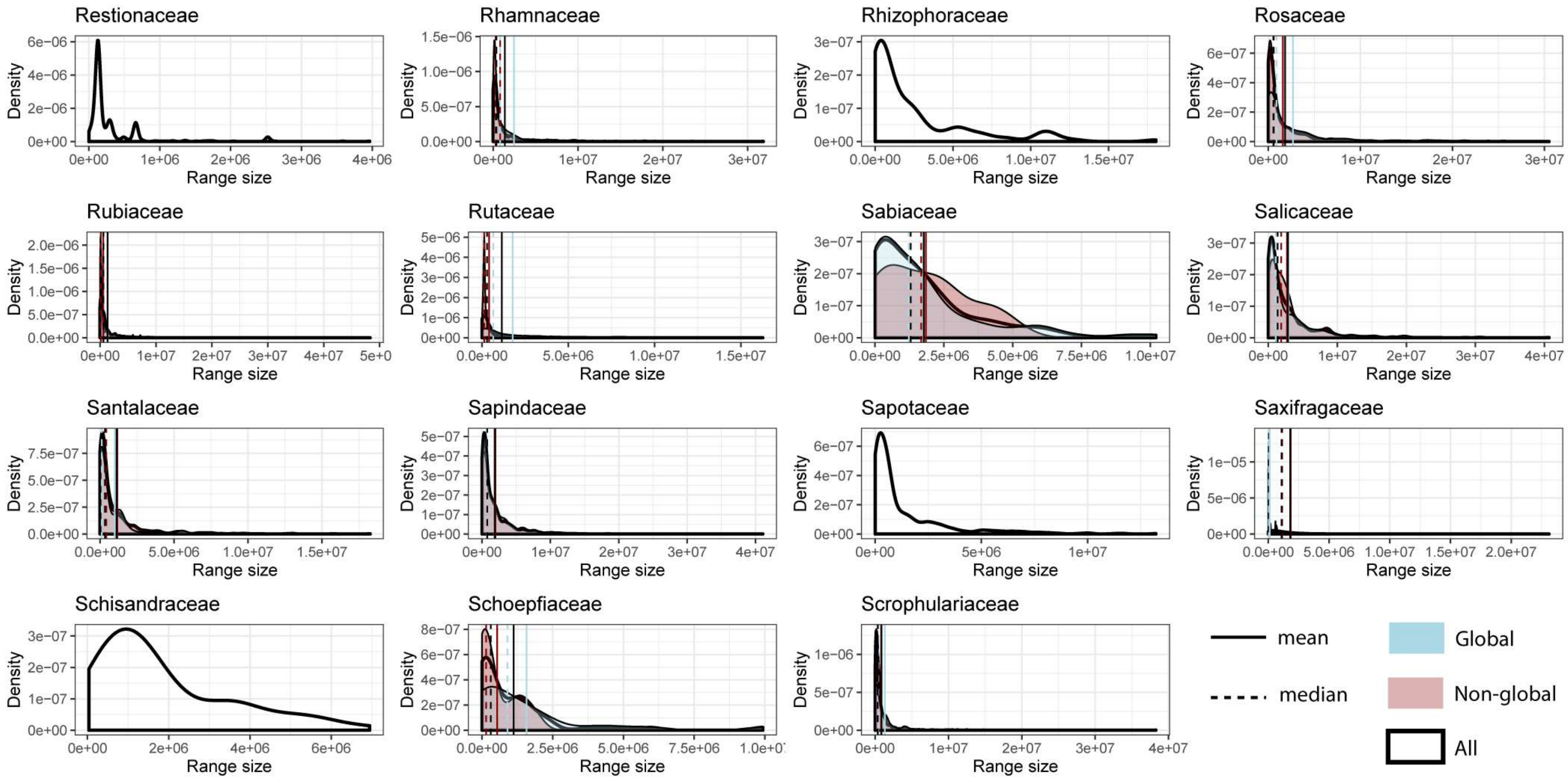
Restionaceae
Rhamnaceae
Rhizophoraceae
Rosaceae
Rubiaceae
Rutaceae
Sabiaceae
Salicaceae
Santalaceae
Sapindaceae
Sapotaceae
Saxifragaceae
Schisandraceae
Schoepfiaceae
Scrophulariaceae
Density
Range size
mean
median
Global
Non-global
All

26

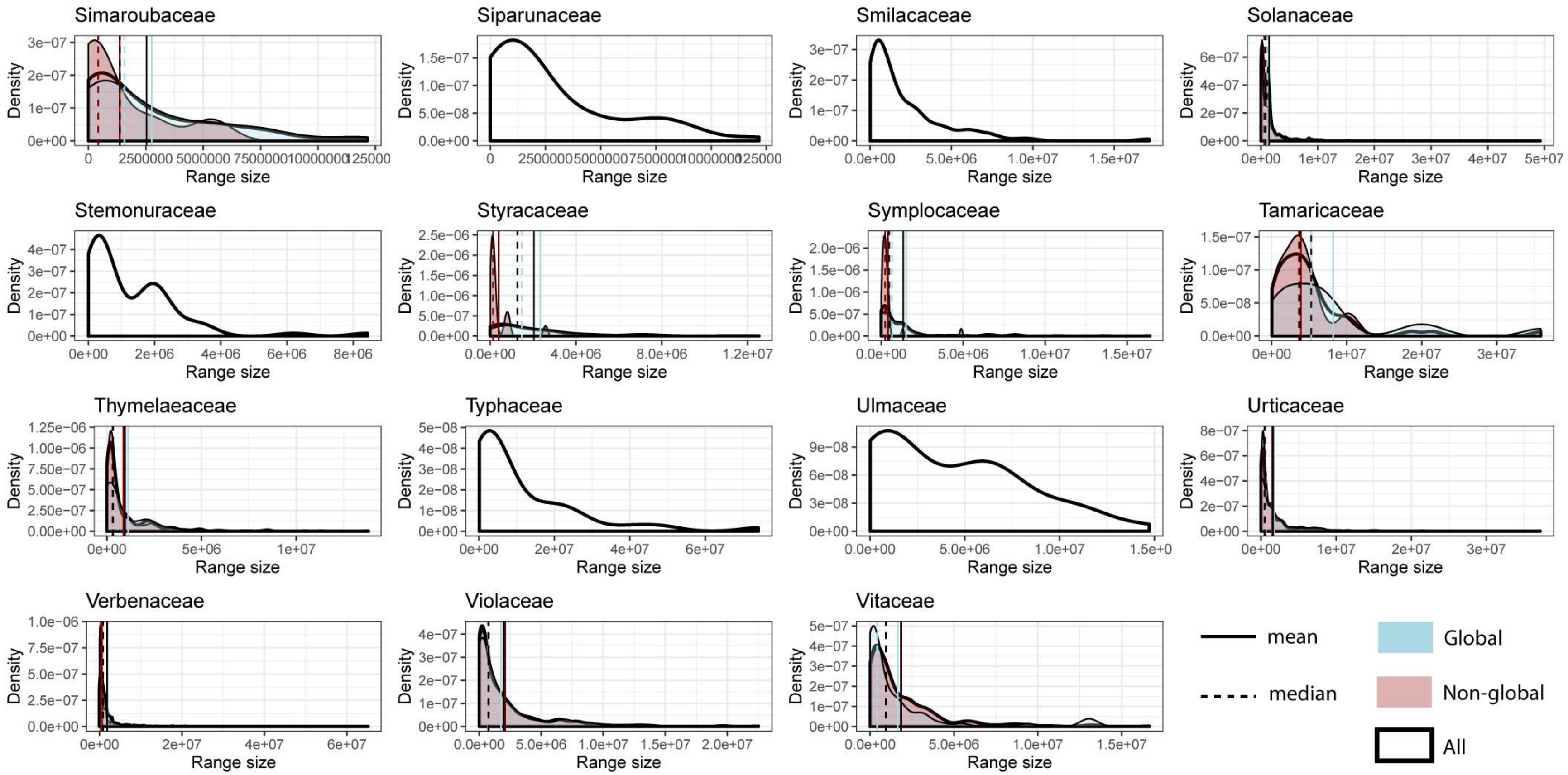
Simaroubaceae
Siparunaceae
Smilacaceae
Solanaceae
Stemonuraceae
Styracaceae
Symplocaceae
Tamaricaceae
Thymelaeaceae
Typhaceae
Ulmaceae
Urticaceae
Verbenaceae
Violaceae
Vitaceae
Density
Range size
mean
median
Global
Non-global
All

27

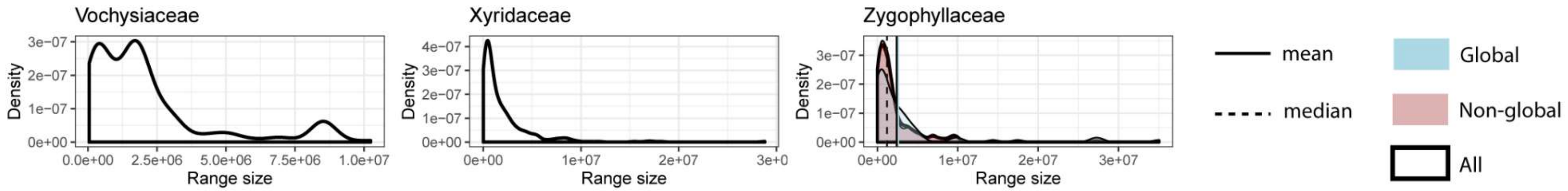
Vochysiaceae
Density
3e−07
2e−07
1e−07
0e+00
0.0e+00
2.5e+06
5.0e+06
7.5e+06
1.0e+07
Range size
Xyridaceae
Density
4e−07
3e−07
2e−07
1e−07
0e+00
0e+00
1e+07
2e+07
3e+0
Range size
Zygophyllaceae
Density
3e−07
2e−07
1e−07
0e+00
0e+00
1e+07
2e+07
3e+07
Range size
mean
median
Global
Non-global
All

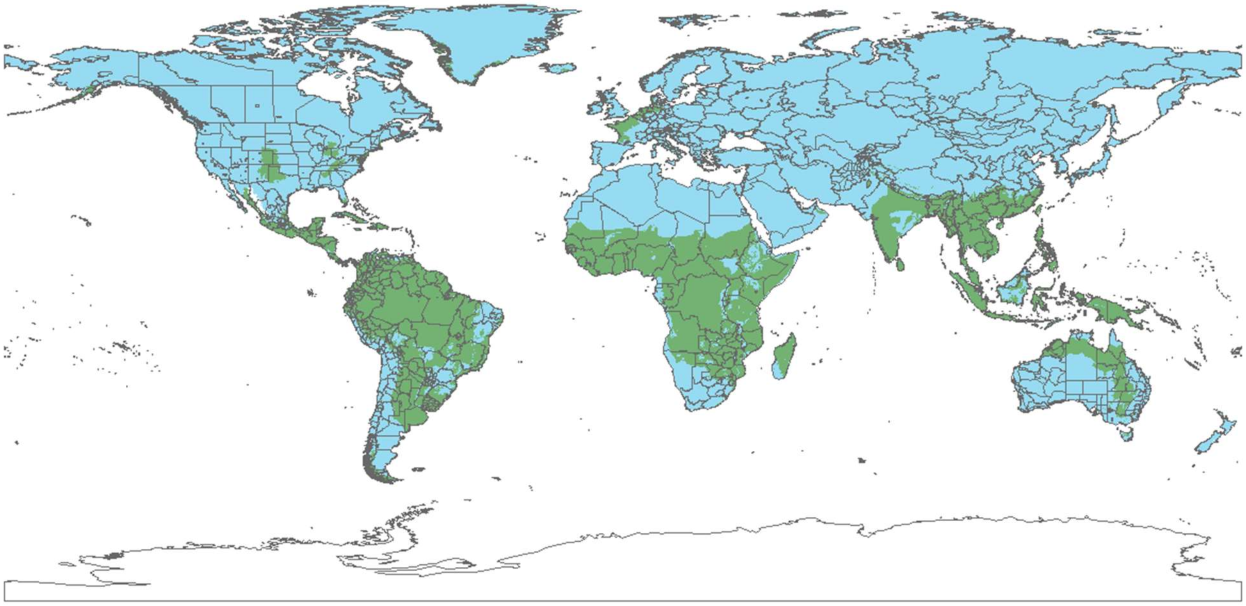

Fig S2: World map including the non-overlapping regions used to calculate range estimates in the analysis. Antarctica and all ice-covered areas are excluded from the calculations. Coloration indicates the tropical and subtropical (green) and temperate (blue) regions following the criteria described in the methods section and the classification by Antonelli et al. (2015). Regions which have temperate and (sub-) tropical climates where assigned to the climate which covers the largest part of the respective region.

Fig. S3: Diagnostic plots to assess chain convergence for the different PGLMM runs for model 1. Each model for 520,000 iterations with a 20,000 iterations burn-in and a thinning interval of 100 and for 3 repetitions.

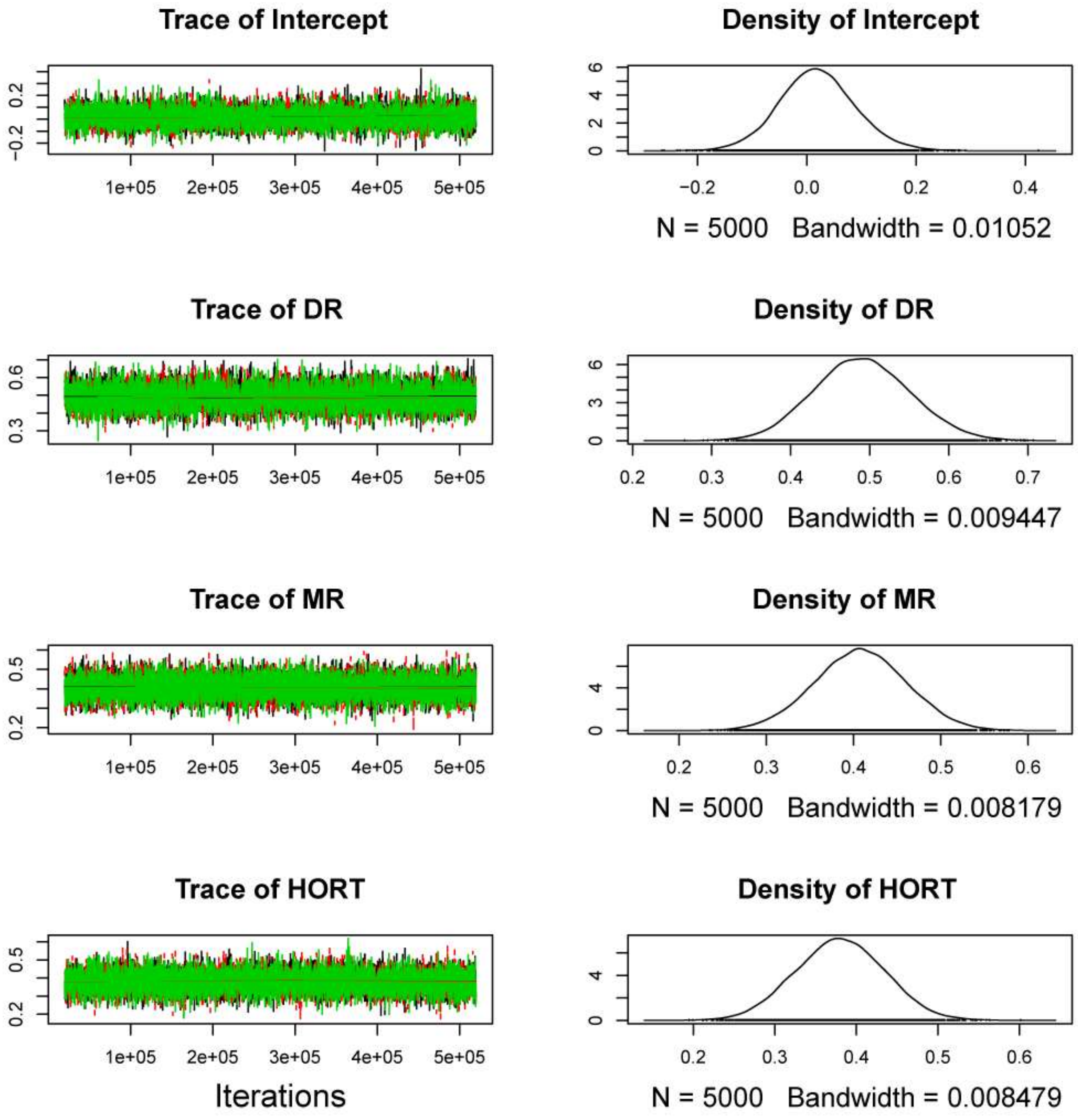

Fig. S4: Diagnostic plots to assess chain convergence for the different PGLMM runs for model 2. Each model for 520,000 iterations with a 20,000 iterations burn-in and a thinning interval of 100 and for 3 repetitions.

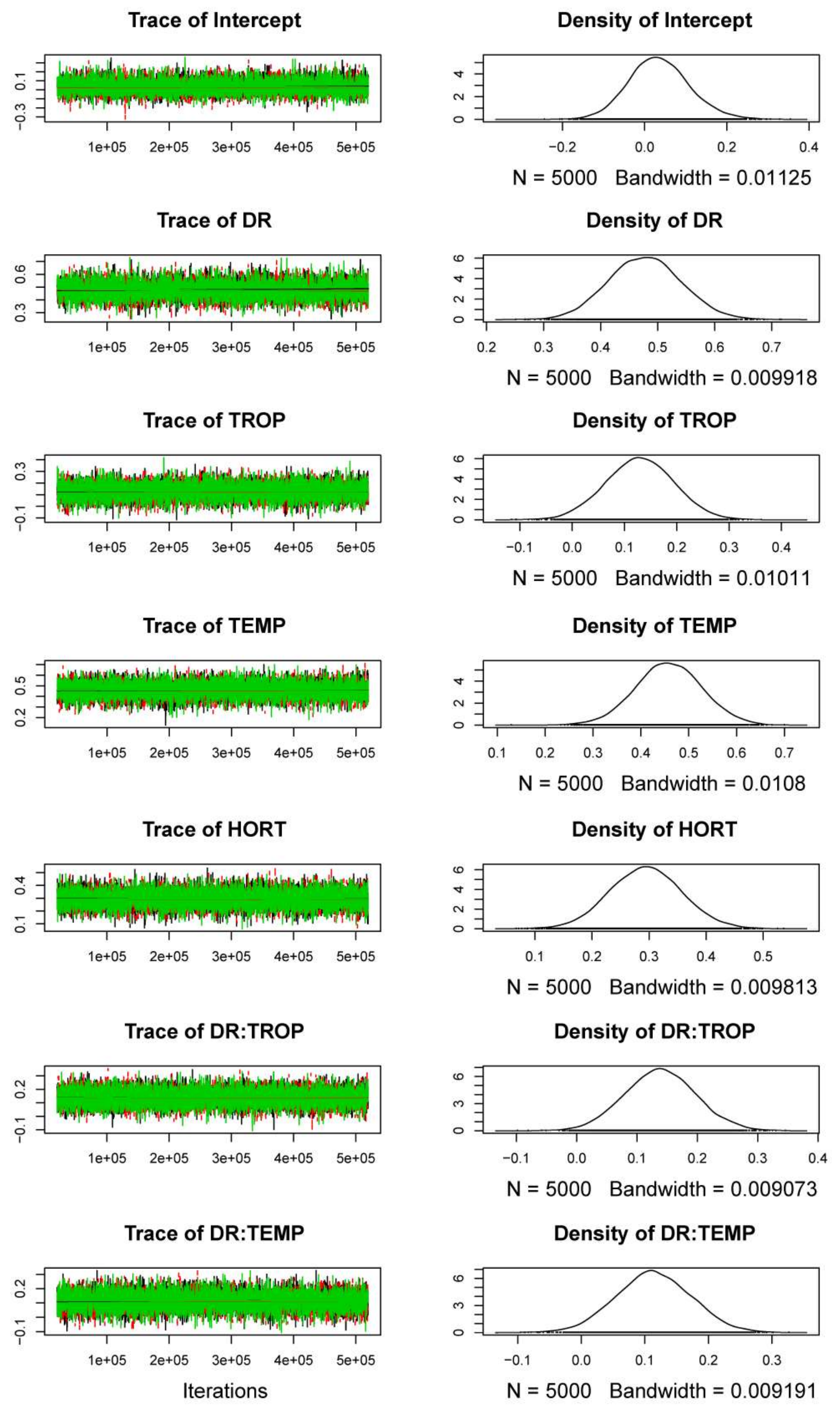

Fig. S5: Diagnostic plots to assess chain convergence for the different PGLMM runs for model 3. Each model for 520,000 iterations with a 20,000 iterations burn-in and a thinning interval of 100 and for 3 repetitions.

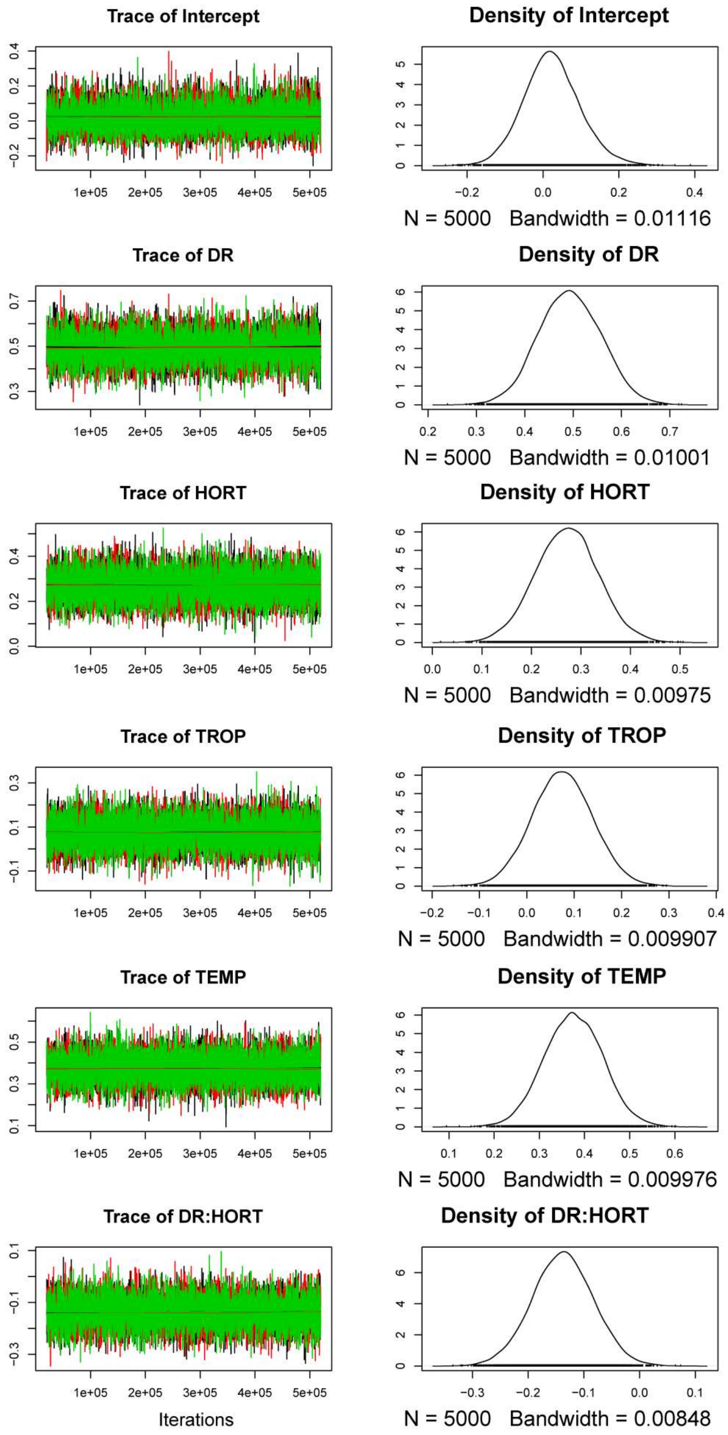

Table S1: Families considered as accepted by The Plant List and that have been excluded from the analysis.

Achatocarpaceae	Eupomatiaceae	Pentadiplandraceae
Acoraceae	Eupteleaceae	Pentaphragmataceae
Acrobolbaceae	Eustichiaceae	Pentaphylacaceae
Adelanthaceae	Exormothecaceae	Penthoraceae
Aextoxicaceae	Fabroniaceae	Peridiscaceae
Akaniaceae	Fissidentaceae	Petenaeaceae
Allisoniaceae	Flagellariaceae	Petermanniaceae
Alseuosmiaceae	Fontinalaceae	Petrosaviaceae
Alstroemeriaceae	Fossombroniaceae	Philesiaceae
Altingiaceae	Fouquieriaceae	Philydraceae
Amblystegiaceae	Funariaceae	Phyllodrepaniaceae
Amborellaceae	Garryaceae	Phyllogoniaceae
Anacampserotaceae	Geissolomataceae	Phyllonomaceae
Anarthriaceae	Gelsemiaceae	Physenaceae
Anastrophyllaceae	Geocalycaceae	Phytolaccaceae
Ancistrocladaceae	Gerrardinaceae	Picramniaceae
Andreaeaceae	Gigaspermaceae	Pilotrichaceae
Andreaeobryaceae	Ginkgoaceae	Pinaceae
Anemiaceae	Gisekiaceae	Plagiochilaceae
Aneuraceae	Gleicheniaceae	Plagiogyriaceae
Anisophylleaceae	Gnetaceae	Plagiotheciaceae
Antheliaceae	Goebeliellaceae	Platanaceae
Anthocerotaceae	Gomortegaceae	Pleurophascaceae
Aphanopetalaceae	Goupiaceae	Pleuroziaceae
Aphloiaceae	Grimmiaceae	Pleuroziopsaceae
Apleniaceae	Grubbiaceae	Plocospermataceae
Apodanthaceae	Gymnomitriaceae	Podocarpaceae
Aponogetonaceae	Gyrostemonaceae	Podostemaceae
Araucariaceae	Gyrothyraceae	Polypodiaceae
Archidiaceae	Halophytaceae	Polytrichaceae
Argophyllaceae	Hanguanaceae	Pontederiaceae
Aristolochiaceae	Haplomitriaceae	Porellaceae
Arnelliaceae	Haptanthaceae	Portulacaceae
Asphodelaceae	Hedwigiaceae	Posidoniaceae
Aspleniaceae	Heliconiaceae	Potamogetonaceae
Asteliaceae	Helicophyllaceae	Pottiaceae
Asteropeiaceae	Helwingiaceae	Prionodontaceae
Atherospermataceae	Herbertaceae	Pseudoditrichaceae
Athyriaceae	Himantandraceae	Pseudolepicoleaceae
Aulacomniaceae	Hookeriaceae	Psilotaceae

Austrobaileyaceae
Aytoniaceae
Balanopaceae
Balanophoraceae
Balantiopsaceae
Barbeuiaceae
Barbeyaceae
Bartramiaceae
Basellaceae
Bataceae
Berberidopsidaceae
Betulaceae
Biebersteiniaceae
Bixaceae
Blandfordiaceae
Blasiaceae
Blechnaceae
Bonnetiaceae
Boryaceae
Brachytheciaceae
Brassicaceae
Brevianthaceae
Bruchiaceae
Bryaceae
Bryobartramiaceae
Bryoxiphiaceae
Burseraceae
Butomaceae
Buxbaumiaceae
Byblidaceae
Cabombaceae
Calomniaceae
Calycanthaceae
Calymperaceae
Calypogeiaceae
Campynemataceae
Canellaceae
Cannaceae
Cardiopteridaceae
Caricaceae
Carlemanniaceae
Caryocaraceae
Catagoniaceae
Huaceae
Hydatellaceae
Hydnoraceae
Hydrocharitaceae
Hydroleaceae
Hydrostachyaceae
Hylocomiaceae
Hymenophyllaceae
Hypnaceae
Hypnodendraceae
Hypodematiaceae
Hypopterygiaceae
Hypoxidaceae
Iridaceae
Irvingiaceae
Isoetaceae
Iteaceae
Ixioliriaceae
Ixonanthaceae
Jackiellaceae
Joinvilleaceae
Jubulaceae
Jubulopsaceae
Juncaceae
Juncaginaceae
Jungermanniaceae
Kirkiaceae
Koeberliniaceae
Krameriaceae
Lacistemataceae
Lactoridaceae
Lanariaceae
Lardizabalaceae
Lejeuneaceae
Lembophyllaceae
Lepicoleaceae
Lepidobotryaceae
Lepidolaenaceae
Lepidoziaceae
Leptodontaceae
Lepyrodontaceae
Leskeaceae
Leucodontaceae
Pteridaceae
Pterigynandraceae
Pterobryaceae
Ptilidiaceae
Ptychomitriaceae
Ptychomniaceae
Quillajaceae
Racopilaceae
Radulaceae
Rafflesiaceae
Rapateaceae
Regmatodontaceae
Rhabdodendraceae
Rhabdoweisiaceae
Rhachidosoraceae
Rhachitheciaceae
Rhacocarpaceae
Rhipogonaceae
Rhizogoniaceae
Ricciaceae
Riellaceae
Rigodiaceae
Roridulaceae
Rousseaceae
Ruppiaceae
Rutenbergiaceae
Saccolomataceae
Salvadoraceae
Salviniaceae
Sarcobataceae
Sarcolaenaceae
Sarraceniaceae
Saururaceae
Scapaniaceae
Scheuchzeriaceae
Schistochilaceae
Schistostegaceae
Schizaeaceae
Schlegeliaceae
Sciadopityaceae
Scorpidiaceae
Selaginellaceae
Seligeriaceae

Catoscopiaceae
Centrolepidaceae
Centroplacaceae
Cephalotaceae
Cephaloziaceae
Cephaloziellaceae
Ceratophyllaceae
Cercidiphyllaceae
Chaetophyllopsaceae
Chonecoleaceae
Cibotiaceae
Cinclidotaceae
Circaeasteraceae
Cistaceae
Cleomaceae
Cleveaceae
Climaciaceae
Colchicaceae
Columelliaceae
Commelinaceae
Conocephalaceae
Coriariaceae
Corsiaceae
Corsiniaceae
Corynocarpaceae
Costaceae
Crossosomataceae
Cryphaeaceae
Ctenolophonaceae
Culcitaceae
Cupressaceae
Curtisiaceae
Cyatheaceae
Cycadaceae
Cyclanthaceae
Cymodoceaceae
Cynomoriaceae
Cyrillaceae
Cyrtopodaceae
Cystopteridaceae
Cytinaceae
Daltoniaceae
Daphniphyllaceae
Leucomiaceae
Limeaceae
Limnanthaceae
Linderniaceae
Lindsaeaceae
Lomariopsidaceae
Lophiocarpaceae
Lophocoleaceae
Lophoziaceae
Lowiaceae
Loxsomataceae
Lunulariaceae
Lycopodiaceae
Lygodiaceae
Makinoaceae
Marattiaceae
Marchantiaceae
Marsileaceae
Martyniaceae
Mastigophoraceae
Matoniaceae
Mayacaceae
Meesiaceae
Melianthaceae
Mesoptychiaceae
Metaxyaceae
Meteoriaceae
Metteniusaceae
Metzgeriaceae
Microtheciellaceae
Misodendraceae
Mitrastemonaceae
Mitteniaceae
Mizutaniaceae
Mniaceae
Monocleaceae
Monosoleniaceae
Montiniaceae
Moringaceae
Muntingiaceae
Musaceae
Myodocarpaceae
Myricaceae
Sematophyllaceae
Serpotortellaceae
Setchellanthaceae
Simmondsiaceae
Sladeniaceae
Sorapillaceae
Sphaerocarpaceae
Sphaerosepalaceae
Sphagnaceae
Sphenocleaceae
Spiridentaceae
Splachnaceae
Splachnobryaceae
Stachyuraceae
Staphyleaceae
Stegnospermataceae
Stemonaceae
Stereophyllaceae
Stilbaceae
Strasburgeriaceae
Strelitziaceae
Stylidiaceae
Surianaceae
Takakiaceae
Talinaceae
Tapisciaceae
Targioniaceae
Taxaceae
Tecophilaeaceae
Tectariaceae
Tetrachondraceae
Tetramelaceae
Tetrameristaceae
Tetraphidaceae
Thamnobryaceae
Theaceae
Theliaceae
Thelypteridaceae
Thomandersiaceae
Thuidiaceae
Thurniaceae
Thyrsopteridaceae
Ticodendraceae

Dasypogonaceae
Datiscaceae
Davalliaceae
Degeneriaceae
Dendrocerotaceae
Dennstaedtiaceae
Diapensiaceae
Dicksoniaceae
Dicnemonaceae
Dicranaceae
Didiereaceae
Dioncophyllaceae
Dipentodontaceae
Diphysciaceae
Diplaziopsidaceae
Dipteridaceae
Dirachmaceae
Disceliaceae
Ditrichaceae
Doryanthaceae
Drosophyllaceae
Dryopteridaceae
Ecdeiocoleaceae
Echinodiaceae
Emblingiaceae
Encalyptaceae
Entodontaceae
Ephedraceae
Ephemeraceae
Equisetaceae
Erpodiaceae
Eucommiaceae
Euphorbiaceae
Euphroniaceae
Myriniaceae
Myrothamnaceae
Myuriaceae
Nartheciaceae
Neckeraceae
Nelumbonaceae
Neotrichocoleaceae
Nepenthaceae
Nephrolepidaceae
Neuradaceae
Nitrariaceae
Nothofagaceae
Notothyladaceae
Octoblepharaceae
Oedipodiaceae
Olacaceae
Oleaceae
Oleandraceae
Oncothecaceae
Onocleaceae
Ophioglossaceae
Opiliaceae
Orthorrhynchiaceae
Orthotrichaceae
Osmundaceae
Oxymitraceae
Paeoniaceae
Pallaviciniaceae
Pandaceae
Pandanaceae
Paracryphiaceae
Paulowniaceae
Pelliaceae
Penaeaceae
Timmiaceae
Tofieldiaceae
Torricelliaceae
Tovariaceae
Trachypodaceae
Treubiaceae
Trichocoleaceae
Trichotemnomataceae
Trigoniaceae
Triuridaceae
Trochodendraceae
Tropaeolaceae
Vahliaceae
Vandiemeniaceae
Velloziaceae
Vetaformaceae
Viridivelleraceae
Vivianiaceae
Wardiaceae
Welwitschiaceae
Wiesnerellaceae
Winteraceae
Woodsiaceae
Xeronemataceae
Zamiaceae
Zingiberaceae
Zosteraceae

Table S2: Overview of the number of species records for each family and used within the study to calculate mean species range sizes. “Overall family richness” provides the number of species per family based on the Plant List (see methods section). “Number of species used” provides the number of species per family with information in the GIFT database. “Number of species with non-global coverage” provides the number of species without global coverage of the species distribution in the GIFT database. “Number of species with global coverage” provides the number of species that are assumed to have global coverage of the species distribution in the GIFT database.

Family	Overall family richness	Number of species used	Number of species with non-global coverage	Number of species with global coverage
Acanthaceae	3947	2683	2161	522
Achariaceae	101	90	8	82
Actinidiaceae	176	144	57	87
Adoxaceae	194	167	78	89
Aizoaceae	2271	1545	1540	5
Alismataceae	120	114	1	113
Amaranthaceae	2052	1622	1420	202
Amaryllidaceae	2258	2141	8	2133
Anacardiaceae	701	554	109	445
Annonaceae	2106	1773	340	1433
Apiaceae	3257	2311	2083	228
Apocynaceae	5556	4275	2030	2245
Aquifoliaceae	480	453	70	383
Araceae	3368	3308	14	3294
Araliaceae	1533	1504	70	1434
Arecaceae	2522	2461	8	2453
Asparagaceae	2929	2847	23	2824
Asteraceae	32913	22055	17729	4326
Balsaminaceae	488	283	275	8
Begoniaceae	1601	1579	5	1574
Berberidaceae	755	469	381	88
Bignoniaceae	852	842	7	835
Boraginaceae	2686	2107	1515	592
Bromeliaceae	3320	3269	6	3263
Brunelliaceae	60	60	0	60
Bruniaceae	92	44	44	0
Burmanniaceae	163	162	0	162
Buxaceae	122	107	28	79
Cactaceae	2715	1548	912	636
Calceolariaceae	281	252	116	136

Calophyllaceae	127	119	5	114
Calyceraceae	56	46	43	3
Campanulaceae	2385	2338	5	2333
Cannabaceae	102	94	9	85
Capparaceae	449	336	118	218
Caprifoliaceae	857	665	553	112
Caryophyllaceae	2456	1701	1413	288
Casuarinaceae	91	91	0	91
Celastraceae	1168	955	446	509
Chloranthaceae	71	67	16	51
Chrysobalanaceae	535	534	1	533
Clethraceae	85	84	6	78
Clusiaceae	1047	940	45	895
Combretaceae	480	350	124	226
Connaraceae	267	202	84	118
Convolvulaceae	1296	1072	708	364
Cornaceae	124	76	2	74
Crassulaceae	1670	1062	967	95
Cucurbitaceae	965	730	617	113
Cunoniaceae	245	225	9	216
Cyperaceae	5784	5426	37	5389
Dichapetalaceae	196	177	73	104
Dilleniaceae	219	193	75	118
Dioscoreaceae	653	643	2	641
Dipterocarpaceae	147	145	1	144
Droseraceae	210	180	0	180
Ebenaceae	777	743	2	741
Elaeagnaceae	108	87	16	71
Elaeocarpaceae	644	604	11	593
Elatinaceae	57	45	41	4
Ericaceae	3544	2749	2062	687
Eriocaulaceae	1206	1187	2	1185
Erythroxylaceae	267	230	20	210
Escalloniaceae	55	47	16	31
Fabaceae	24505	16335	9846	6489
Fagaceae	1101	926	6	920
Frankeniaceae	73	69	64	5
Gentianaceae	1682	1274	949	325
Geraniaceae	841	626	556	70
Gesneriaceae	3122	1889	1245	644
Goodeniaceae	329	319	292	27
Grossulariaceae	195	159	140	19
Gunneraceae	69	62	0	62

Haemodoraceae	101	101	0	101
Haloragaceae	92	84	80	4
Hamamelidaceae	99	84	20	64
Hernandiaceae	51	49	27	22
Humiriaceae	63	62	8	54
Hydrangeaceae	237	207	186	21
Hypericaceae	584	403	266	137
Icacinaceae	212	159	77	82
Juglandaceae	89	71	5	66
Lamiaceae	7866	7310	97	7213
Lauraceae	2978	2687	133	2554
Lecythidaceae	341	339	2	337
Lentibulariaceae	312	265	230	35
Liliaceae	746	704	16	688
Linaceae	213	170	140	30
Loasaceae	314	255	190	65
Loganiaceae	351	305	152	153
Loranthaceae	886	654	549	105
Lythraceae	604	497	268	229
Magnoliaceae	250	238	2	236
Malpighiaceae	1301	1094	450	644
Malvaceae	4465	3657	1735	1922
Marantaceae	569	561	5	556
Marcgraviaceae	137	121	82	39
Melanthiaceae	181	171	1	170
Melastomataceae	4079	3028	957	2071
Meliaceae	669	607	38	569
Menispermaceae	448	370	289	81
Menyanthaceae	55	42	39	3
Molluginaceae	103	93	86	7
Monimiaceae	134	114	13	101
Montiaceae	113	95	94	1
Moraceae	1217	1090	122	968
Myristicaceae	170	165	11	154
Myrtaceae	5970	5750	17	5733
Nyctaginaceae	450	365	210	155
Nymphaeaceae	70	48	43	5
Ochnaceae	560	499	148	351
Onagraceae	832	635	546	89
Orchidaceae	27541	26569	161	26408
Orobanchaceae	1613	1325	1220	105
Oxalidaceae	601	520	406	114
Papaveraceae	920	572	541	31

Passifloraceae	932	801	477	324
Pedaliaceae	67	47	39	8
Peraceae	116	108	0	108
Phrymaceae	199	134	131	3
Phyllanthaceae	2099	2047	9	2038
Picrodendraceae	96	96	0	96
Piperaceae	2658	2239	1059	1180
Pittosporaceae	170	150	79	71
Plantaginaceae	1614	1314	1115	199
Plumbaginaceae	635	386	262	124
Poaceae	11554	10895	133	10762
Polemoniaceae	562	380	361	19
Polygalaceae	1163	920	637	283
Polygonaceae	1384	1092	836	256
Primulaceae	2788	1954	1001	953
Proteaceae	1252	1181	843	338
Putranjivaceae	216	216	0	216
Ranunculaceae	2377	1708	1602	106
Resedaceae	51	45	41	4
Restionaceae	482	527	1	526
Rhamnaceae	839	712	446	266
Rhizophoraceae	142	129	5	124
Rosaceae	4828	2988	2244	744
Rubiaceae	13673	13394	62	13332
Rutaceae	1730	1467	665	802
Sabiaceae	116	113	20	93
Salicaceae	1269	1057	335	722
Santalaceae	992	742	575	167
Sapindaceae	1751	1570	359	1211
Sapotaceae	1343	1251	5	1246
Saxifragaceae	775	602	586	16
Schisandraceae	73	73	4	69
Schoepfiaceae	51	48	21	27
Scrophulariaceae	1576	1226	1100	126
Simaroubaceae	121	90	14	76
Siparunaceae	61	53	0	53
Smilacaceae	261	257	1	256
Solanaceae	2678	2101	1243	858
Stemonuraceae	71	66	0	66
Styracaceae	133	128	16	112
Symplocaceae	210	201	32	169
Tamaricaceae	79	54	35	19
Thymelaeaceae	938	769	489	280

Typhaceae	65	51	0	51
Ulmaceae	64	59	3	56
Urticaceae	1465	1033	622	411
Verbenaceae	1035	950	34	916
Violaceae	806	613	417	196
Vitaceae	985	596	532	64
Vochysiaceae	215	212	3	209
Xyridaceae	387	385	0	385
Zygophyllaceae	211	183	151	32

Table S3: Full dataset used in the analysis including 168 plant families. Overall and naturalized species richness values were obtained from The Plant List (www.theplantlist.org) and the GloNAF database (Pyšek et al. 2017), respectively. Diversification rates were estimated using the protocol by Magallon & Sanderson (2001) using ages from the global angiosperm phylogeny of (Magallón et al. 2015). Range size estimates were calculated based on information provided by the GIFT database (Weigelt et al. 2019). Horticulturally used species richness is based on (van Kleunen *et al.* 2018). Naturalization success was calculated based on naturalized and native species richness and the number of regions where species of that family are naturalized in. For more details on data sources see Methods section.

**Family**	**Overall richness**	**Naturalized richness**	**Family age**	**Horticulturally used species**	**Diversification rate**	**Mean family range**	**Mean tropical family range**	**Mean temperate family range**	**Naturalization success**
Poaceae	11554	1399	59	5230	0.12	7431169	2736869	4925109	3374
Asteraceae	32913	1678	49	12436	0.164	2183784	319438	2450465	1150
Fabaceae	24505	1388	92	10741	0.085	2942011	1276061	2145193	994
Amaranthaceae	2052	277	64	1070	0.083	5541078	662344	2187274	971
Rosaceae	4828	635	99	2908	0.062	3306593	135829	3700681	774
Plantaginaceae	1614	260	49	1227	0.105	3476802	314239	3157044	649
Solanaceae	2678	281	67	1233	0.084	2559439	1166215	1429931	587
Polygonaceae	1384	196	68	970	0.073	5901944	474878	2916780	541
Caryophyllaceae	2456	244	70	1514	0.079	4342565	119753	2343096	491
Onagraceae	832	186	73	743	0.061	4230202	676603	2440949	379
Convolvulaceae	1296	167	67	668	0.073	5728474	2092118	1562066	330
Lamiaceae	7866	409	40	3588	0.166	3319817	1324903	2081419	297
Malvaceae	4465	293	60	1874	0.102	3164015	1687028	1429642	262
Boraginaceae	2686	225	88	1487	0.063	2898983	679388	2407593	231
Apiaceae	3257	211	58	1769	0.1	2751501	134858	2898026	199
Papaveraceae	920	91	113	514	0.04	2953518	41243	3127476	174
Cyperaceae	5784	386	55	2621	0.115	8708216	4077165	5230818	168
Caprifoliaceae	857	108	71	545	0.063	2998566	205847	2158614	159
Geraniaceae	841	93	102	553	0.043	2761895	122256	3020361	144
Ranunculaceae	2377	177	80	1615	0.068	4078125	61324	2863369	124
Asparagaceae	2929	178	62	1919	0.091	2283842	1000365	1418328	117

Crassulaceae	1670	148	95	1589	0.052	1915158	59911	3076688	115
Oxalidaceae	601	60	57	292	0.072	3717357	1053338	2025863	112
Amaryllidaceae	2258	161	62	1465	0.087	2399549	633300	1918659	108
Verbenaceae	1035	77	40	385	0.117	4141667	2718038	1522513	108
Salicaceae	1269	86	74	614	0.065	4809527	1396888	5079436	93
Cucurbitaceae	965	57	57	422	0.08	3695282	787330	1815567	80
Tamaricaceae	79	21	54	43	0.04	5753978	140742	2989917	61
Alismataceae	120	33	72	106	0.035	9628190	4568170	5390623	59
Bignoniaceae	852	65	47	421	0.095	3727171	3893637	255025	54
Adoxaceae	194	33	71	157	0.042	4519433	764283	4695326	52
Ulmaceae	64	13	79	53	0.025	4923677	1413867	10741725	52
Moraceae	1217	73	69	644	0.07	4176160	3667228	706136	52
Lythraceae	604	53	73	227	0.057	3500663	1594624	2081635	47
Cactaceae	2715	114	29	2655	0.188	1110297	576113	2446067	46
Cannabaceae	102	12	73	84	0.033	8724927	7170520	2312293	46
Scrophulariaceae	1576	82	49	666	0.104	1483192	174211	3410712	45
Resedaceae	51	9	50	35	0.036	2802319	86360	2387509	45
Linaceae	213	24	87	147	0.036	2541583	553118	2463838	42
Araceae	3368	136	129	1901	0.045	2220467	1905725	545451	40
Passifloraceae	932	51	89	419	0.051	2598484	1481321	1290959	40
Myrtaceae	5970	174	86	2713	0.075	1117943	1096475	930726	38
Apocynaceae	5556	123	52	2584	0.121	2584566	1694749	1457257	37
Anacardiaceae	701	40	52	375	0.083	3672728	2133189	1822374	35
Acanthaceae	3947	143	43	953	0.138	2321192	696172	1943330	35
Typhaceae	65	11	76	34	0.026	22244424	4252217	20969228	35
Juglandaceae	89	19	69	72	0.033	3947782	714394	6477036	34
Sapindaceae	1751	69	79	707	0.065	3257463	1820195	2027036	31
Nyctaginaceae	450	22	64	209	0.06	2872897	1538106	1866315	30
Nymphaeaceae	70	17	125	56	0.016	9175142	1091852	1964898	29
Haloragaceae	92	15	72	65	0.032	4490194	389391	2184727	29

Urticaceae	1465	57	69	451	0.073	2957784	1286517	1660970	28
Casuarinaceae	91	10	85	85	0.027	1021473	991335	1378389	28
Hypericaceae	584	54	68	301	0.06	2761007	1071078	1997917	28
Grossulariaceae	195	18	86	155	0.035	3206998	97594	2934489	25
Rubiaceae	13673	195	68	3017	0.107	2490540	2073191	513634	24
Hydrangeaceae	237	26	91	203	0.035	1880905	202041	3548988	24
Elaeagnaceae	108	10	65	48	0.038	5647303	873358	9823022	24
Arecaceae	2522	133	98	2289	0.056	1501010	1586897	103878	24
Campanulaceae	2385	95	76	1167	0.072	2088381	776901	1434536	23
Aizoaceae	2271	73	75	2187	0.073	920344	79662	4152405	21
Molluginaceae	103	10	73	36	0.033	6705911	657466	3435471	21
Violaceae	806	41	83	458	0.053	3581462	883151	1845908	20
Polemoniaceae	562	37	83	497	0.046	1771314	68244	3796523	19
Rutaceae	1730	54	67	879	0.077	1879548	1403308	2206331	18
Orobanchaceae	1613	69	36	869	0.142	2876954	169660	2902442	18
Vitaceae	985	44	121	379	0.038	3015407	644095	2381172	17
Primulaceae	2788	73	87	1095	0.065	2343578	861431	1914523	16
Araliaceae	1533	51	60	598	0.084	1505548	1199714	331090	15
Phrymaceae	199	14	33	193	0.091	2519637	142988	3581216	14
Zygophyllaceae	211	12	61	116	0.051	5100209	1126930	2677814	13
Rhamnaceae	839	38	70	487	0.064	2570625	1421217	3360445	13
Balsaminaceae	488	15	50	148	0.077	1714405	38001	3572782	13
Berberidaceae	755	30	80	435	0.054	1581177	269736	3121087	12
Meliaceae	669	21	69	368	0.061	3716802	3700614	912789	12
Liliaceae	746	39	44	581	0.098	2970204	283709	3074730	12
Cornaceae	124	11	93	119	0.028	5674184	2340229	3122082	11
Pittosporaceae	170	15	63	133	0.046	1527728	613659	2195407	11
Combretaceae	480	26	90	221	0.043	4751172	3265700	744210	10
Phyllanthaceae	2099	43	84	529	0.064	2941666	2639814	344712	10
Plumbaginaceae	635	31	68	276	0.061	2495735	141230	2372316	10
Fagaceae	1101	40	98	595	0.048	1864694	1071008	1018770	9

Simaroubaceae	121	7	67	51	0.038	4311212	3542877	1172594	8
Dioscoreaceae	653	20	73	190	0.058	3089097	2922185	306738	8
Gentianaceae	1682	41	52	763	0.099	2826632	551040	2275638	7
Montiaceae	113	9	43	111	0.058	3746604	251721	3644398	7
Ericaceae	3544	79	69	1975	0.085	1937853	317288	3037639	7
Magnoliaceae	250	22	109	243	0.03	1745899	1655845	230647	6
Menyanthaceae	55	6	68	36	0.027	6143567	1271068	2210935	6
Celastraceae	1168	33	93	408	0.051	3368803	1628142	1582661	6
Pedaliaceae	67	6	22	52	0.092	4434899	238132	2811919	6
Saxifragaceae	775	30	86	640	0.051	3058355	3480	2937092	5
Proteaceae	1252	40	106	1043	0.046	574147	309304	3677168	4
Elatinaceae	57	9	74	34	0.026	5345917	152574	2340875	4
Buxaceae	122	6	85	55	0.03	1813032	372933	1988687	3
Annonaceae	2106	17	94	581	0.057	2212406	1948912	562660	2
Begoniaceae	1601	34	37	798	0.136	1058242	1069557	65813	2
Lauraceae	2978	20	105	756	0.054	1799156	1555304	793061	2
Melastomataceae	4079	45	73	693	0.083	1932928	1755073	715442	2
Piperaceae	2658	33	66	535	0.085	1968221	1619349	855663	2
Marantaceae	569	28	38	244	0.106	2907070	3080768	40747	2
Polygalaceae	1163	17	96	276	0.05	2056729	944301	2060885	2
Orchidaceae	27541	111	109	10870	0.073	1580370	1367661	523697	1
Aquifoliaceae	480	11	64	233	0.061	1710361	1363214	1332298	1
Sapotaceae	1343	23	94	655	0.052	2169420	2194488	170417	1
Melanthiaceae	181	9	84	163	0.035	2530346	629672	2672526	1
Thymelaeaceae	938	15	60	428	0.076	1281157	421269	2321101	1
Frankeniaceae	73	3	54	36	0.039	1851746	62830	2735150	1
Escalloniaceae	55	3	93	38	0.02	1379283	1196591	1991023	1
Bromeliaceae	3320	41	76	2303	0.077	1021650	1231005	100035	1
Droseraceae	210	10	77	190	0.039	3032137	1606181	3133638	1
Clusiaceae	1047	14	91	313	0.051	1833878	1880307	322972	1

Gunneraceae	69	3	105	35	0.02	738118	756669	218863	1
Rhizophoraceae	142	7	73	45	0.037	4014525	4567452	657730	1
Ebenaceae	777	11	87	752	0.05	2246540	1916198	195266	1
Calceolariaceae	281	6	49	126	0.069	874843	736669	1308931	1
Chrysobalanaceae	535	12	55	102	0.073	2980044	3242133	21544	1
Capparaceae	449	10	44	203	0.086	4064248	2386406	1412779	1
Styracaceae	133	6	53	77	0.05	2571888	1884095	1467744	1
Actinidiaceae	176	5	85	95	0.034	1516315	708988	1665750	1
Lentibulariaceae	312	11	43	217	0.08	4947375	1578963	1616901	1
Menispermaceae	448	9	90	153	0.043	3005973	1480045	1193939	1
Malpighiaceae	1301	20	74	201	0.066	2100512	1926524	804443	0
Lecythidaceae	341	11	100	183	0.036	2154710	2482020	36259	0
Haemodoraceae	101	4	44	81	0.054	573342	407449	2239230	0
Ochnaceae	560	8	92	86	0.044	2891760	2329716	532214	0
Xyridaceae	387	7	77	87	0.048	2872374	2698342	448341	0
Loasaceae	314	9	91	171	0.038	1097597	468574	2385097	0
Calyceraceae	56	2	49	30	0.038	985023	378178	3164100	0
Dilleniaceae	219	6	123	60	0.026	2811832	2159759	488875	0
Achariaceae	101	6	94	40	0.025	2447010	2178226	667280	0
Smilacaceae	261	7	44	121	0.075	3174967	2651072	967313	0
Hamamelidaceae	99	4	96	63	0.025	2330809	1351288	1449193	0
Santalaceae	992	9	81	224	0.057	2049838	685694	2344258	0
Clethraceae	85	3	79	25	0.028	1253650	1190419	328448	0
Loganiaceae	351	4	60	123	0.06	2891058	1995693	620717	0
Goodeniaceae	329	4	57	195	0.062	1059291	77582	2828532	0
Myristicaceae	170	3	85	82	0.034	3311887	3461708	87663	0
Gesneriaceae	3122	17	49	1249	0.118	621568	306122	1953755	0
Chloranthaceae	71	2	135	20	0.015	1599652	1084047	690299	0

Hernandiaceae	51	3	91	27	0.02	2585857	2104761	1027569	0
Eriocaulaceae	1206	7	77	175	0.062	2247745	2098312	227038	0
Elaeocarpaceae	644	4	82	167	0.051	1622681	1548173	607099	0
Peraceae	116	2	74	20	0.034	2381493	2379079	332694	0
Calophyllaceae	127	1	80	24	0.033	2364152	2481430	158513	0
Schisandraceae	73	1	110	58	0.019	2234030	1461236	833119	0
Erythroxylaceae	267	3	73	59	0.046	2496718	2583228	498559	0
Schoepfiaceae	51	1	58	15	0.031	1490369	1118650	1138540	0
Icacinaceae	212	1	69	52	0.045	2963968	2463742	817744	0
Symplocaceae	210	2	81	63	0.038	2443197	1869326	846522	0
Loranthaceae	886	3	68	155	0.066	1875344	658453	2093567	0
Burmanniaceae	163	1	73	26	0.039	3114775	3228175	277222	0
Putranjivaceae	216	1	59	67	0.053	1691501	1650756	125517	0
Cunoniaceae	245	1	81	76	0.04	975092	1169573	907677	0
Brunelliaceae	60	0	85	22	0.023	745342	1342902	0	0
Bruniaceae	92	0	78	30	0.03	139511	0	4496110	0
Connaraceae	267	0	57	54	0.058	3111272	2116232	533967	0
Dichapetalaceae	196	0	52	36	0.058	2398063	1597330	596007	0
Dipterocarpaceae	147	0	36	111	0.077	2405002	2058883	14389	0
Humiriaceae	63	0	104	7	0.019	3664325	3977478	120701	0
Marcgraviaceae	137	0	55	32	0.048	1610990	1426066	791156	0
Monimiaceae	134	0	91	37	0.029	1695934	1825354	668012	0
Picrodendraceae	96	0	84	44	0.028	846249	1043530	666229	0
Restionaceae	482	0	67	197	0.058	355555	126611	1293474	0
Sabiaceae	116	0	128	44	0.02	2266380	1445502	1154949	0
Siparunaceae	61	0	92	18	0.021	3265920	4144984	43018	0
Stemonuraceae	71	0	74	16	0.028	1803754	1704180	268412	0
Vochysiaceae	215	0	86	10	0.036	2367811	2683433	107019	0

Table S4: Pearson correlation between all predictor variable combinations. Variables were standardized if appropriate and all variables were standardized beforehand. See methods section for details.

	Mean Family Range	Horticultural Use	Mean Tropical Family Range	Mean Temperate Family Range
Diversification Rate	0.01	-0.03	-0.13	0.06
Mean Family Range		0.10	0.37	0.31
Horticultural Use			-0.36	0.42
Mean Tropical Family Range				-0.42

Table S5: Gelman-Rubin diagnostic to assess chain convergence for all three PGLMM models. Values below 1.1 are indicate an appropriate level of chain convergence. Reported is the point estimate of the multivariate potential scale reduction factor (multivariate psrf).

	**multivariate psrf**
Model 1	1.04
Model 2	1.05
Model 3	1.04

Table S6: Model selection results for model 1. Abbreviations are: DR = diversification rate; MR = mean family range; HORT = horticultural use.

**Model**	**Intercept**	**DR**	**MR**	**HORT**	**logLik**	**DIC**	**delta**
Success ~ DR + MR	0.04	0.49	0.43		45.85	-141	0
Success ~ DR	0.03	0.48			-8.87	-39	102
Success ~ DR + MR + HORT	0.02	0.50	0.40	0.38	-109.33	237	378
Success ~ DR + HORT	0.01	0.48		0.42	-157.41	329	470
Random term (all models):	~Family						

Table S7. Results of the three generalized linear mixed models. Abbreviations are: DR:TROP = interaction term between diversification rate and mean tropical family range; DR:TEMP = interaction term between diversification rate and mean temperate family range; DR:HORT = interaction term between diversification rate and horticultural use. Estimates highlighted in bold indicate significant terms.

	Model 1				Model 2				Model 3			
Predictors	*Estimates*	*std.*	*Statistic*	*p*	*Estimates*	*std.*	*Statistic*	*p*	*Estimates*	*std.*	*Statistic*	*p*
Intercept	0.03	0.07	0.51	0.613	0.04	0.07	0.50	0.617	0.01	0.07	0.20	0.843
Diversification rate	**0.49**	**0.06**	**8.77**	**<0.001**	**0.48**	**0.06**	**8.16**	**<0.001**	**0.50**	**0.06**	**8.53**	**<0.001**
Mean Range	**0.39**	**0.05**	**7.50**	**<0.001**								
Horticultural use	**0.37**	**0.05**	**6.84**	**<0.001**	**0.29**	**0.06**	**4.66**	**<0.001**	**0.27**	**0.06**	**4.31**	**<0.001**
Tropical Range					0.12	0.06	1.80	0.072	0.06	0.06	0.95	0.341
Temperate Range					**0.45**	**0.07**	**6.50**	**<0.001**	**0.36**	**0.06**	**5.62**	**<0.001**
DR : TROP					**0.14**	**0.06**	**2.41**	**0.016**				
DR : TEMP					**0.12**	**0.06**	**2.07**	**0.039**				
DR : HORT									**-0.15**	**0.05**	**-2.84**	**0.005**
Observations	168				168				168			
R^2 conditional	0.604				0.606				0.607			
R^2 marginal	0.544				0.520				0.525			

References

Antonelli, A., Zizka, A., Silvestro, D., Scharn, R., Cascales –Miñana, B. & Bacon, C.D. (2015) An engine for global plant diversity: highest evolutionary turnover and emigration in the American tropics. *Front. Genet.*, 6: 130.

Magallón, S., Gómez-Acevedo, S., Sánchez-Reyes, L.L. & Hernández-Hernández, T. (2015). A metacalibrated time-tree documents the early rise of flowering plant phylogenetic diversity. *New Phytol.*, 207, 437–453.

Magallon, S. & Sanderson, M.J. (2001). Absolute Diversification Rates in Angiosperm Clades. *Evolution (N. Y).*, 55, 1762–1780.

Pyšek, P., Pergl, J., Essl, F., Lenzner, B., Dawson, W., Kreft, H., Weigelt, P., Winter, M., Kartesz, J., Nishino, M., *et al.* (2017). Naturalized alien flora of the world: species diversity, taxonomic and phylogenetic patterns, geographic distribution and global hotspots of plant invasion. *Preslia*, 89, 203–274.

van Kleunen, M., Essl, F., Pergl, J., Brundu, G., Carboni, M., Dullinger, S., Early, R., González-Moreno, P., Groom, Q.J., Hulme, P.E., *et al.* (2018). The changing role of ornamental horticulture in alien plant invasions. *Biol. Rev.*, 93, 1421–1437.

Weigelt, P., König, C. & Kreft, H. (2019). GIFT – A Global Inventory of Floras and Traits for macroecology and biogeography. *J. Biogeogr.*, 1–28.
